# A centralised immunogen approach to develop a more broadly protective modified live porcine reproductive and respiratory syndrome virus 1 vaccine candidate

**DOI:** 10.1038/s41541-025-01192-z

**Published:** 2025-06-21

**Authors:** Rory C. F. de Brito, Yashar Sadigh, Joseph Bowman, Stephanie Clive, Ben Jackson, Miriam Pedrera, Fraser Crofts, Matthieu Bernard, Fabian Z. X. Lean, Alejandro Núñez, Julian Seago, Jean-Pierre Frossard, Simon P. Graham

**Affiliations:** 1 The Pirbright Institute, Woking, UK; 2 Virology Department, Animal and Plant Health Agency, Addlestone, UK; 3 Pathology and Animal Sciences Department, Animal and Plant Health Agency, Addlestone, UK; 4 Present Address: College of Engineering, Environment & Science School of Science, Coventry University, Coventry, UK; 5 Present Address: Centro de Investigación en Sanidad Animal (CISA), Instituto Nacional de Investigación y Tecnología Agraria y Alimentaria (INIA), Consejo Superior de Investigaciones Científicas (CSIC), Valdeolmos, Madrid, Spain; 6 Present Address: Department of Pathobiology and Population Sciences, The Royal Veterinary College, North Mymms, UK

**Keywords:** Microbiology, Vaccines, Live attenuated vaccines

## Abstract

More efficacious vaccines are required to improve control of porcine reproductive and respiratory syndrome viruses (PRRSV). One strategy that has shown promise is the use of centralized antigens, generated from consensus sequence data. Here, we evaluated the consensus sequence approach to develop a PRRSV-1 modified live virus (MLV) vaccine candidate, ‘EU-PRRSV-Con’. EU-PRRSV-Con strain was engineered by inserting consensus sequence open-reading frames encoding envelope proteins of 67 PRRSV-1 strains into an attenuated PRRSV-1 strain backbone. EU-PRRSV-Con was evaluated in pigs and benchmarked against a licensed MLV vaccine. Efficacy was assessed against three different PRRSV-1 isolates. Neutralizing antibodies were elicited by EU-PRRSV-Con, which were more reactive than those induced by the licensed MLV. EU-PRRSV-Con provided better levels of protection (reduced viral loads and lung pathology) than the licensed MLV, although the efficacy against a divergent PRRSV-1 subtype 3 strain was more limited. These data support the development of EU-PRRSV-Con as a vaccine that may aid control of PRRSV-1.

## Introduction

Porcine reproductive and respiratory syndrome virus (PRRSV) is a small, enveloped virus with a single-stranded positive-sense RNA genome, belonging to the genus *Betaarterivirus* within the family *Arteriviridae* (order *Nidovirales*)^1^. There are currently two recognised species of PRRSV; PRRSV-1 (*Betaarterivirus europensis*), which emerged from Western Europe, and PRRSV-2 (*Betaarterivirus americense*) that emerged from North America^2,3^. Both species have now spread near worldwide and PRRSV is endemic in most major swine-producing countries^4^. PRRSV-1 and -2 share approximately 60% nucleotide similarity, with both species exhibiting similar disease phenotypes and clinical symptoms in infected pigs^5^. Infection is typically characterised by reproductive failure in sows and respiratory disease in pigs of all ages. PRRSV also enhances susceptibility to secondary bacterial and viral infections, leading to more severe disease and increased mortality^6^. Consequently, PRRSV is a major threat to animal welfare and global food security, responsible for large economic losses, estimated to cost the USA around US$ 600 million every year^7,8^. This impact has become even more significant due to the rapid evolution of PRRSV, resulting in the emergence of increasingly divergent and pathogenic strains, thus posing a major challenge to the control of the virus through vaccination^5^.

Inactivated and modified live (MLV) virus vaccines have been widely used to control PRRSV. While MLVs confer clinical protection against genetically related PRRSV strains, they provide variable protection against divergent strains, highlighting the urgent need for the development of vaccines capable of providing broader protection^9^. Inactivated PRRSV vaccines have proven to be inadequate in providing immunity against both homologous and heterologous strains, as demonstrated by a lack of PRRSV-specific neutralising antibodies^10^ and weak cell-mediated immune responses^11^. It is thought that PRRSV has evolved strategies to evade the immune system by focusing the response on non-neutralising decoy epitopes and through glycan shielding of neutralising epitopes^12^. As a consequence, neutralising antibodies (nAbs), which are considered an important component of the PRRSV immune response, are typically unable to provide broad protection^6^. Moreover, analysis of several PRRSV isolates reveals the presence of hypervariable regions within the surface glycoproteins, leading to antigenic variation^13^. This evidence highlights that there is a gap of knowledge of PRRSV epitopes that are common to all PRRSV strains, further pointing to the need for new vaccination approaches that can generate more broadly protective immune responses. One possible strategy that has been explored to combat the substantial genetic diversity of PRRSV is the use of centralised sequences, based on aligning PRRSV genomes and then selecting the most common nucleotide found at each position^14^. As such, these consensus vaccine sequences exhibit shorter genetic distances to wild-type viruses^15^. Previous studies have shown that consensus sequence vaccines could effectively address the issue of genetic diversity^16^. For example, vaccines against HIV-1 and influenza virus, which have been designed based on this approach, have been shown to elicit enhanced immune responses compared to vaccines based on naturally occurring sequences^17^.

Using the consensus sequence approach, a synthetic PRRSV-2 vaccine strain, known as PRRSV-Con, was designed based on the full-genome sequence of 59 PRRSV-2 strains and constructed using reverse genetics^14^. This synthetic strain was found to confer significantly higher levels of heterologous protection in pigs compared to a reference wild-type PRRSV-2 strain^14^. Recently, Li et al.^18^ designed and evaluated a chimeric PRRSV-1 vaccine based on ORF2–6 consensus sequence based on 14 representative, mostly Chinese, PRRSV-1 strains. The chimeric vaccine conferred protection against a Chinese subtype 1 PRRSV-1 challenge strain^18^, further demonstrating the potential of the consensus sequence approach for the development of novel PRRSV vaccines^18,19^. This study, therefore, aimed to construct and evaluate a recombinant PRRSV-1 vaccine candidate, EU-PRRSV-Con, designed using the consensus sequence approach. Since the key targets of nAbs are the major (GP5/M) and minor envelope glycoprotein (GP2/GP3/GP4) complexes^20^, the consensus sequences for these open reading frames (ORFs) were generated from 67 PRRSV-1 strains and inserted into the genome of an attenuated PRRSV-1 strain (Olot/91). The stability and replication of EU-PRRSV-Con were assessed in vitro, and its immunogenicity and efficacy were assessed in pigs, benchmarking this against a commercial MLV. Three animal studies were performed to assess protection against three PRRSV-1 field isolates. Animals were assessed for clinical signs and lung pathology and reverse transcriptase quantitative polymerase chain reaction (RT-qPCR) was used to assess viraemia levels and viral shedding from serum and swab samples, respectively, as well as viral loads present in a selection of key tissues. In addition, assays were conducted to assess the antibody and T cell responses elicited by EU-PRRSV-Con.

## Results

### Design and construction of the synthetic EU-PRRSV-Con vaccine candidate

Sequence data for ORFs encoding envelope proteins from PRRSV-1 strains were collated to design consensus sequence ORFs 2–6. From each full genome sequence, the specific ORF2a, ORF2b, ORF3, ORF4, ORF5, ORF5a and ORF6 sequences were identified and translated into the corresponding amino acid sequences. The resulting GP2, E, GP3, GP4, GP5, ORF5a protein, and M protein sequences were aligned, and the consensus sequence was identified for each ORF, based on the most prevalent amino acid at each position (Supplementary Fig. 1A–G, respectively). The consensus amino acid sequence for each ORF was compared to the corresponding sequence deduced for the PRRSV-1 Olot/91 strain. Where differences were identified, the nucleotide sequence was altered to encode the desired amino acid instead of that originally present in the PRRSV-1 Olot/91 strain. To avoid inadvertently affecting viral protein expression through the effects of codon pair bias^21^, the replacement nucleotide codons were chosen from other existing PRRSV-1 sequences in the alignment. As some changes were required in the genome areas where overlaps occur between two ORFs, nucleotide changes were checked to ensure that the resulting amino acid sequences for both ORFs were still as intended. In only one case was this not possible— position 258 of the GP3 protein. The change from leucine (L) to phenylalanine (F) was not possible because of the resulting change in the GP4 protein. The L to F substitution was semi-conservative, as both are hydrophobic and nonpolar, but leucine is aliphatic while phenylalanine is aromatic. A total of 96 nucleotide changes were made in EU-PRRSV-Con versus PRRSV-1 Olot/91. Targeted, silent nucleotide mutations were then introduced to disrupt specific restriction sites, maintain ORFs and facilitate the rescue of infectious virus that was cell culture-adapted for MARC-145 cells. The poly-A tail was extended to enable virus rescue, and an upstream CMV promoter and a downstream ribozyme site were added to enable authentic cap production during transcription and 3’ untranslated processing, respectively. The phylogenetic relationship of the final genetic sequence for EU-PRRSV-Con (AC# PV146404) to the 67 sequences used in the analysis (as well as a representative PRRSV-2 sequence) is shown in the Supplementary Fig. 2.

### EU-PRRSV-Con vaccine candidate shows in vitro replication properties comparable to the parental PRRSV-1 Olot/91 strain

**Fig. 1:**
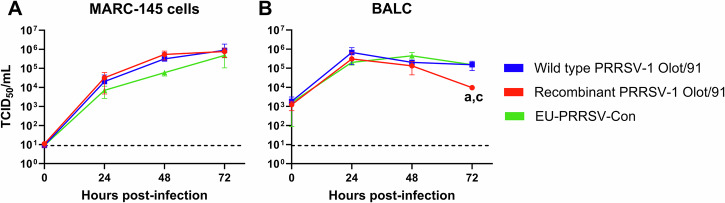
Assessment of EU-PRRSV-Con replication in MARC-145 cells and porcine BALC. Replication kinetics of EU-PRRSV-Con, wild type and recombinant PRRSV-1 Olot/91 in MARC-145 cells (**A**) and BALC (**B**) cultures following infection with a multiplicity of infection of 0.1. Infectious titres in culture supernatants were determined by subsequent titration on MARC-145 cells. Datapoints represent the mean of technical triplicates and error bars represent the standard deviation (SD). Significant differences (*p* < 0.05) are described by letters where a: significant difference from wild type PRRSV-1 Olot/91, and c: significant difference from EU-PRRSV-Con. The dashed horizontal line indicates the assay limit of detection.

The replication of EU-PRRSV-Con was assessed in vitro in MARC-145 cells and porcine bronchoalveolar lavage cells (BALC) and compared to wild type and recombinant forms of PRRSV-1 Olot/91, which formed the backbone of EU-PRRSV-Con. Like wild type and recombinant PRRSV-1 Olot/91, the titres of EU-PRRSV-Con were broadly comparable between MARC-145 cells and BALC (<1log_10_) (Supplementary Fig. 3). Multistep growth curve analysis of EU-PRRSV-Con in both MARC-145 cells and BALC showed comparable replication kinetics with the parental PRRSV-1 Olot/91 viruses (Fig. 1). The only significant difference observed was lower recombinant PRRSV-1 Olot/91 titres in BALC 72 h post-infection, compared to both other viruses.

To assess the genetic stability of EU-PRRSV-Con in cell culture, whole genome sequence analysis was performed on virus, which had been serially passaged 10 times on MARC-145 cells. All three replicate samples (R1, R2 and R3) produced near-complete sequences for the EU-PRRSV-Con genome. All samples were missing less than 20 base pairs at the extreme 5’ end of the genome, and as this is non-coding, this was not considered in the analysis. Average coverage ranged from 190 read depth for R1 to 8128 read depth for R2. The three mutations which correlate with adaption of PRRSV-1 strains to MARC-145 cell adaptation, i.e., GP2 V88F, M94I and F95L^22^ were retained in all three samples. Single-nucleotide polymorphisms (SNPs) were identified in all three samples (Supplementary Table 1). When considering major synonymous SNPs (occurring in >50% of aligned reads), there were 11 unique differences in the three sample sequences compared to the reference sequence, each appearing in only a single sample, apart from one. These were seen twice for R3 in nsp2 (pp1a) with a separate mutation in R1. The mutations seen in nsp2 are not unexpected, as this is known to be the most variable protein in PRRSV^23^. In pp1b there were four mutations in R1, where one was shared with R3, and another unique mutation was seen in R2. For R1, there were two mutations in nsp9 (RNA-dependent polymerase) and one each in nsp10 (helicase) and nsp12 (involved in sub-genomic mRNA generation). The mutation shared with R3 was in nsp10. In the structural protein encoding ORFs, R3 had a unique synonymous mutation in GP5 and R1 had another unique synonymous mutation in the M protein. The remaining 11 SNPs were synonymous changes in all three samples compared to the reference sequence. The majority of these remaining SNPs were in the non-structural proteins. Three mutations were in the structural protein encoding ORFs and were spread between GP2, GP5 and the M protein.

### Evaluation of the immunogenicity and efficacy of the EU-PRRSV-Con vaccine candidate

To evaluate the safety, immunogenicity, and efficacy of EU-PRRSV-Con, three vaccination and challenge studies were performed (Table 1). Pigs were vaccinated with EU-PRRSV-Con or a commercial PRRSV-1 MLV (Porcilis PRRS MLV (MSD Animal Health), herein referred to as ‘benchmark MLV’) or received a placebo inoculation. Thirty-five days post-vaccination, pigs were challenged by intranasal inoculation with PRRSV-1 subtype 1 strains 215-06 (study 1) and 21301-19 (study 2), and subtype 3 strain SU1-Bel (study 3). The % identities between the two vaccines and three challenge viruses are shown in Supplementary Table 2. For studies 2 and 3, two additional groups were vaccinated but not challenged to assess vaccine virus in blood and tissues in the absence of challenge. Pigs were euthanized on 7-, 21- and 22-days post-challenge (DPC) to assess lung pathology, viral loads in tissues and local immune responses. Back titrations on MARC-145 cells and BALC were performed to confirm of vaccine and challenge virus doses, respectively (Supplementary Table 3). For all three studies, the inoculation doses of both vaccine and challenge viruses were within (±) 1 log_10_ of the target dose of 10^5^ TCID_50_.

**Table 1:** Evaluation of the immunogenicity and efficacy of EU-PRRSV-Con: Study design

Group	Days post-immunisation			
	0	35	42	56/57
	Vaccination	Challenge	Post-mortem analysis	Post-mortem analysis
**1**	Benchmark MLV PRRSV-1	Yes	*n* = 3	*n* = 6
**2**	EU-PRRSV-Con	Yes	*n* = 3	*n* = 6
**3**	None	Yes	*n* = 3	*n* = 6
4*	Benchmark MLV PRRSV-1	None	None	*n* = 6
5*	EU-PRRSV-Con	None	None	*n* = 6

*Studies 2 and 3 only.

### Vaccination of pigs with EU-PRRSV-Con does not induce clinical signs and, compared to a benchmark MLV, provides improved clinical protection against virulent PRRSV-1 challenge

**Fig. 2:**
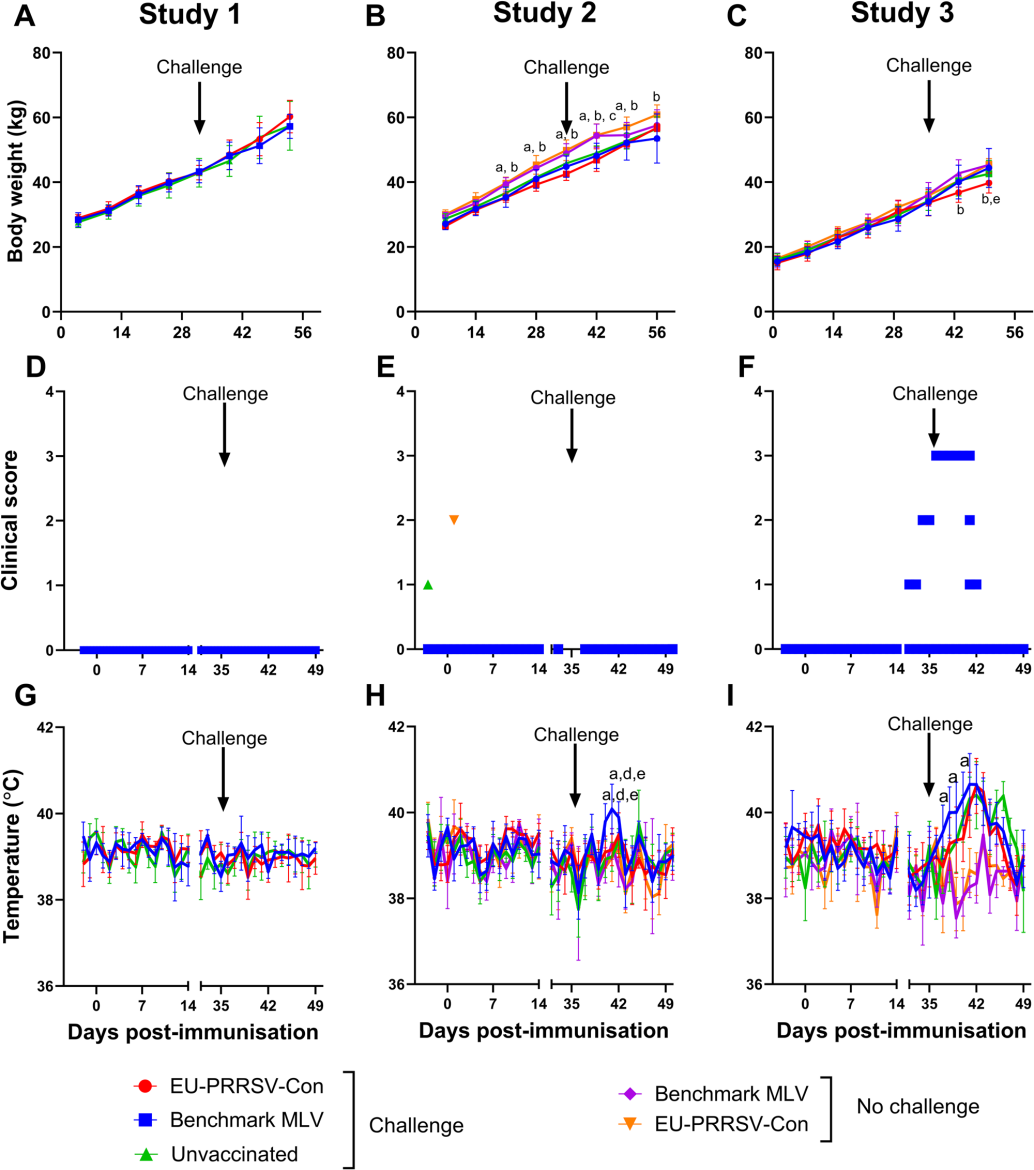
Weight gain, clinical signs, and rectal temperatures following immunisation and challenge in all three studies. On day 0, pigs were vaccinated with either EU-PRRSV-Con or benchmark MLV PRRS or were unvaccinated. On day 35 post-vaccination, pigs were challenged with the PRRSV-1 strains 215-06 (Study 1), 21301-19 (Study 2) or SU1-Bel (Study 3). In studies 2 and 3, groups of vaccinated pigs were left unchallenged. Following immunisation and challenge, animals were weighed on a weekly basis (**A**–**C**) and clinical signs (**D**–**F**) and rectal temperatures (**G**–**I**) were measured daily for fourteen days. Data presented as mean body weight and rectal temperatures (error bars represent the SD) or datapoints representing the clinical scores for individual pigs. Significances (*p* < 0.05) are described by letters where a: significant difference from EU-PRRSV-Con/challenged; b: significant difference from benchmark MLV/challenged; c: significant difference from unvaccinated/challenged; d: significant difference from EU-PRRSV-Con/no challenge; e: significant difference from benchmark MLV/no challenge.

In all three studies, pigs were weighed on a weekly basis. In study 1, no significant differences were observed between treatment groups at any of the timepoints (Fig. 2A). In study 2, from day 21, animals from both vaccinated and unchallenged groups had a significantly higher mean body weight compared to the EU-PRRSV-Con vaccinated and challenged (Fig. 2B). Also, the mean body weight was higher on EU-PRRSV-Con vaccinated and unchallenged group compared to benchmark MLV vaccinated. On day 42, all groups that were challenged demonstrated significantly lower body weights in comparison to the vaccinated and unchallenged groups. Lower body weights were measured in both vaccinated and challenged groups, which differed from EU-PRRSV-Con vaccinated and unchallenged on day 49. Then on the last time point EU-PRRSV-Con vaccinated and unchallenged had higher body weight mean compared to the benchmark MLV vaccinated and challenged group. In study 3, the EU-PRRSV-Con vaccinated and challenged group had a lower mean body weight than the benchmark MLV vaccinated and challenged group (day 43 and 50) and the EU-PRRSV-Con vaccinated and unchallenged group (day 50) (Fig. 2C). However, these data may have been skewed by the change in group compositions following the removal of three pigs from each group on day 42.

Animals were scored for clinical signs and rectal temperatures were measured daily for 14 days after vaccination and challenge. In study 1, prior to and post-challenge with PRRSV-1 215-06, no clinical signs were observed in any of the groups (Fig. 2D), and rectal temperatures were stable throughout, which were similar between the groups (Fig. 2G). Likewise, in study 2, following challenge with PRRSV-1 21301-19, all groups showed no clinical signs, however mild clinical signs were observed in one of the animals from the unvaccinated and from the EU-PRRSV-Con vaccinated group on a single day prior to challenge (Fig. 2E). Animals in the benchmark MLV vaccinated group displayed a brief period of elevated rectal temperatures (>40 °C) at day 40 and 41 post-immunisation (days 5 and 6 post-challenge). On day 40, these temperatures were significantly higher in the benchmark MLV/challenge group than the EU-PRRSV-Con/challenge (*p* < 0.01) and unvaccinated groups (*p* < 0.001) (Fig. 2H). Again, on day 41, significantly higher temperatures were measured in the benchmark MLV/challenge group than the EU-PRRSV-Con/challenge and unvaccinated groups (*p* < 0.0001). In study 3, following PRRSV-1 SU1-Bel challenge, three of the pigs from the benchmark MLV/challenge group and one from the unvaccinated group developed typical clinical signs of PRRSV infection, such as reduced eating and interaction, ear necrosis and a reddening of the skin on the ears and body (Fig. 2F). In addition, one of the three pigs from the benchmark MLV/challenge group also developed PRRSV-related clinical signs pre-challenge. Conversely, no clinical signs were observed in any of the EU-PRRSV-Con vaccinated animals. Following challenge, animals in the benchmark MLV, EU-PRRSV-Con and unvaccinated groups showed an increase in rectal temperatures from day 36–42 (Fig. 2I). During this period, higher temperatures were observed in the three challenge groups compared to the no challenge groups, with there being overall higher temperatures in the benchmark MLV group following challenge. At days 37–38 and 40, temperatures in this group were significantly higher than the EU-PRRSV-Con/challenge group (*p* < 0.05 and *p* < 0.01, respectively). However, higher temperatures were observed in both the EU-PRRSV-Con/challenged and unvaccinated/challenged groups following challenge until day 45, after which higher temperatures were observed in unvaccinated/challenged group.

### Compared to a benchmark MLV, EU-PRRSV-Con provides improved protection against lung pathology associated with virulent PRRSV-1 challenge

**Fig. 3:**
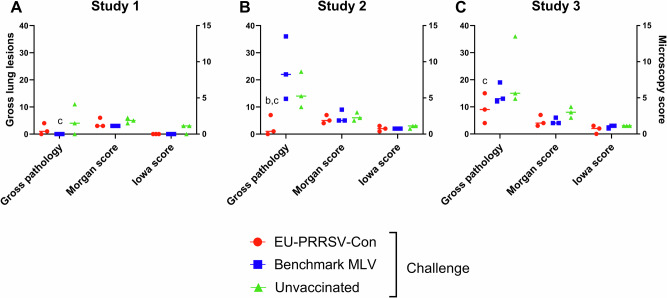
Gross lung pathology, histopathology, and viral immunohistochemistry of lungs post-challenge. Scoring of macroscopic and microscopic lung lesions at 7 DPC was performed blind in study 1 (**A**), 2 (**B**) and 3 (**C**). Each lobe was given a score for lesion coverage, from which each lobe score was weighted based on its relative size and then summed to give the gross lung pathology score. Microscopic lesions were scored using the “Morgan” scoring system on H&E-stained sections and the “Iowa” scoring system, which incorporates the presence of viral antigen, assessed by IHC. Significances (*p* < 0.05) are described by letters where a: significant difference from EU-PRRSV-Con/challenged; b: significant difference from benchmark MLV/challenged; and c: significant difference from unvaccinated/challenged group.

Lungs were assessed macroscopically for lesions alongside with histopathology and viral immunohistochemical (IHC) analyses at day 42 (7 DPC) (Fig. 3). In study 1, no lesions were recorded in any of the benchmark MLV vaccinated animals at 7 DPC and the average lung lesion score in this group was significantly lower (*p* < 0.05) than that in the unvaccinated animals. The low gross lung lesion scores observed in the EU-PRRSV-Con vaccinated group did not differ significantly from the benchmark MLV vaccinated nor unvaccinated groups. Low histopathology scores were obtained in all groups based on Morgan scoring scheme, and only 2/3 pigs in the unvaccinated and challenged group had low Iowa IHC scores characterised by sparse viral antigen (Fig. 3A). In study 2, there were significantly lower gross lesion scores in the EU-PRRSV-Con vaccinated animals than in the benchmark MLV (*p* < 0.001) and unvaccinated groups (*p* < 0.05) at 7 DPC. However, no significant differences were detected between the benchmark MLV and unvaccinated animals. Animals displayed low-moderate histopathology scores using both systems and with no significant differences between treatment groups (Fig. 3B). Likewise, in study 3, there were significantly lower (*p* < 0.05) average lesion scores in the EU-PRRSV-Con animals compared to the unvaccinated animals. Compared to unvaccinated pigs, both EU-PRRSV-Con and benchmark MLV vaccinated pigs displayed significantly lower histopathology scores according to the Morgan scheme (*p* < 0.05). No differences were observed using the Iowa IHC scoring system, as shown in Fig. 3C.

### Compared to a benchmark MLV, vaccination with EU-PRRSV-Con reduces viral loads following heterologous PRRSV-1 challenge

**Fig. 4:**
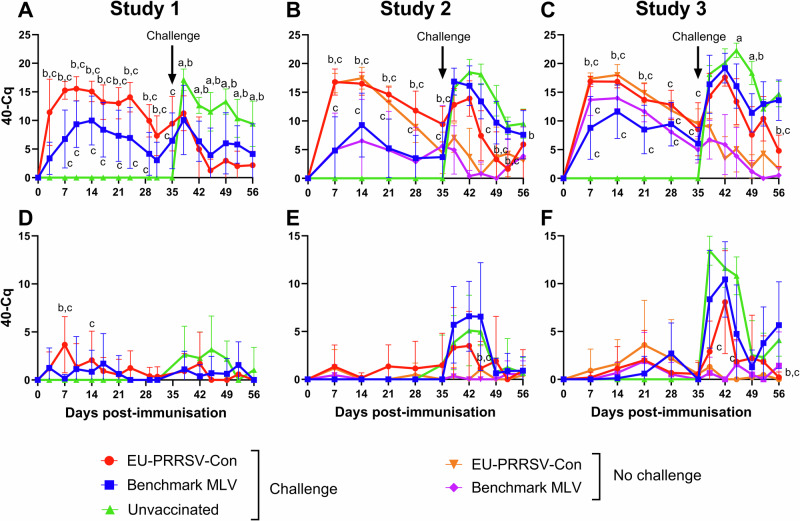
RT-qPCR results of serum and nasal swab samples of each group across all studies. Blood samples and nasal swabs were collected weekly post-vaccination and twice weekly post-challenge. Viraemia (**A**–**C**) and virus shedding (**D**–**F**) were inferred by RT-qPCR. Results are expressed as the mean data for each group at each timepoint and error bars represent the SD. Significances (*p* < 0.05) are described by letters where a: significant difference from EU-PRRSV-Con/challenged; b: significant difference from benchmark MLV/challenged; c: significant difference from unvaccinated/challenged; d: significant difference from EU-PRRSV-Con/no challenge; e: significant difference from benchmark MLV/no challenge.

Nasal swabs and blood samples were collected to infer vaccine and challenge PRRSV-1 shedding and viraemia, respectively, by RT-qPCR (Fig. 4). Analysis of serum during the pre-challenge period using the area under the curve (AUC), revealed increased PRRSV RNA levels in serum in all vaccinated groups, with no viral RNA detected in the unvaccinated groups, and significantly higher levels of RNA observed in the EU-PRRSV-Con immunised animals compared to the benchmark MLV immunised animals across all studies (*p* < 0.05; Supplementary Table 4). In contrast, during the post-challenge period, lower PRRSV RNA levels were observed in both vaccinated and challenged groups compared to the unvaccinated and challenged groups across all three studies (*p* < 0.05; Supplementary Table 4). As expected, there were low levels of viral RNA detected in the no challenge groups following challenge (Fig. 4A–C). In study 1, from day 38 (3 DPC) until the end of the study, there were significantly lower levels of RNAemia in the EU-PRRSV-Con/challenged and benchmark MLV/challenged groups than those that were unvaccinated (*p* < 0.001 and *p* < 0.05, respectively at day 56 (21 DPC) (Fig. 4A). In study 2, by day 49 (14 DPC) until the end of the study, there were significantly lower levels (*p* < 0.05) of viraemia in the EU-PRRSV-Con/challenged than the benchmark MLV/challenged group, which saw similar levels to the unvaccinated animals (Fig. 4B). Furthermore, on day 56 in study 3, significantly lower levels of RNAemia (*p* < 0.0001) were present in the EU-PRRSV-Con/challenged group compared to the benchmark MLV/challenged and unvaccinated/challenged group, however no significant differences were detected between the benchmark MLV/challenged and unvaccinated//challenged groups at the end of the study (Fig. 4C).

RT-qPCR analyses of nasal swab samples showed lower levels of viral RNA, in all groups across the studies, compared to serum samples (Fig. 4D–F). In study 1, no significant differences were observed between treatment groups at any of the timepoints. Similar to the RNAemia results, less viral RNA was shed from the EU-PRRSV-Con/challenged animals post-challenge (Fig. 4D) and in study 2, at day 45, there was significantly less virus shed compared to the benchmark MLV/challenged (*p* < 0.0001) and unvaccinated/challenged (*p* < 0.01) groups (Fig. 4E). As seen with the RNAemia levels in study 3, higher levels of virus shedding were detected in the unvaccinated and benchmark MLV/challenged groups post-challenge compared to the EU-PRRSV-Con/challenged group and at day 56, this was significantly higher in the benchmark MLV/challenged and unvaccinated groups (*p* < 0.001 and *p* < 0.05, respectively) (Fig. 4F). Only in study 3, did AUC analysis show lower nasal shedding in both vaccinated and challenge groups compared to the unvaccinated and challenged group (*p* < 0.05; Supplementary Table 4)

Virus loads in bronchoalveolar lavage fluid (BALF), tracheobronchial lymph node (TBLN) and lung tissues collected post-mortem were inferred by RT-qPCR; (Supplementary Fig. 6. In BALF, in studies 1, similar levels of virus were detected in the benchmark MLV and EU-PRRSV-Con vaccinated and challenged animals at 7 DPC, which significantly differed from unvaccinated and challenge animals (*p* < 0.05). However, on 21/22 DPC significantly lower levels of RNA (*p* < 0.05) in the EU-PRRSV-Con vaccinated and challenged animals were observed compared to unvaccinated or vaccinated with the benchmark MLV, with the complete absence of detectable viral RNA in 3/6 pigs (Supplementary Fig. 6A). However, in study 2 and study 3, no significant differences were detected in BALF between any of the vaccinated/challenged and unvaccinated/challenged groups at either timepoint (Supplementary Fig. 6B, C). In study 1, viral RNA was higher in the lung tissue of the EU-PRRSV-Con/challenged and unvaccinated groups than in the benchmark MLV/challenged group at 7 DPC (*p* < 0.05) (Supplementary Fig. 6D). However, on 21/22 DPC, viral RNA was significantly lower in the EU-PRRSV-Con/challenged group compared to the benchmark MLV/challenged (*p* < 0.05) and unvaccinated/challenged groups (*p* < 0.001). In studies 2 and 3, no differences were observed between the challenged groups in any time point (Supplementary Fig. 6E, F). For the TBLN analyses, in study 1 at 7 DPC viral load in the benchmark MLV/challenged pigs was significantly lower than that in the EU-PRRSV-Con/challenged pigs (*p* < 0.05) (Supplementary Fig. 6G). No significant differences in viral RNA loads between the vaccinated/challenged and unvaccinated groups were detected in either study 2 or 3 (Supplementary Fig. 6H–I). Similar to the RNAemia data, there was a trend towards higher levels of RNA being detected in all three tissue samples from the EU-PRRSV-Con/no challenge groups compared to the benchmark MLV/no challenge groups.

### Neutralising antibodies elicited by EU-PRRSV-Con are more reactive than those induced by the benchmark MLV

Serum antibody responses were assessed using a commercial diagnostic serological PRRSV ELISA test (Supplementary Fig. 7). In study 1, significant antibody responses were detected in day 31 serum samples from both EU-PRRSV-Con and benchmark MLV vaccinated groups when compared to unvaccinated/challenged group (*p* < 0.01). On day 56, antibody levels were significantly higher in the benchmark MLV group compared to both the EU-PRRSV-Con vaccinated (*p* < 0.05) and unvaccinated (*p* < 0.01) groups (Supplementary Fig. 7A). In study 2, day 35 serum samples in all vaccinated groups had antibody levels higher than the unvaccinated group (*p* < 0.01). Whereas, on day 56, only the antibody levels in the EU-PRRSV-Con/challenged and benchmark MLV/challenged groups were significantly higher than the unvaccinated group (*p* < 0.05) (Supplementary Fig. 7B). In study 3, antibody levels were higher in serum at day 35 in all vaccinated groups compared to the unvaccinated group (*p* < 0.001), but no significant differences were observed between the groups’ sera at day 56.

**Fig. 5:**
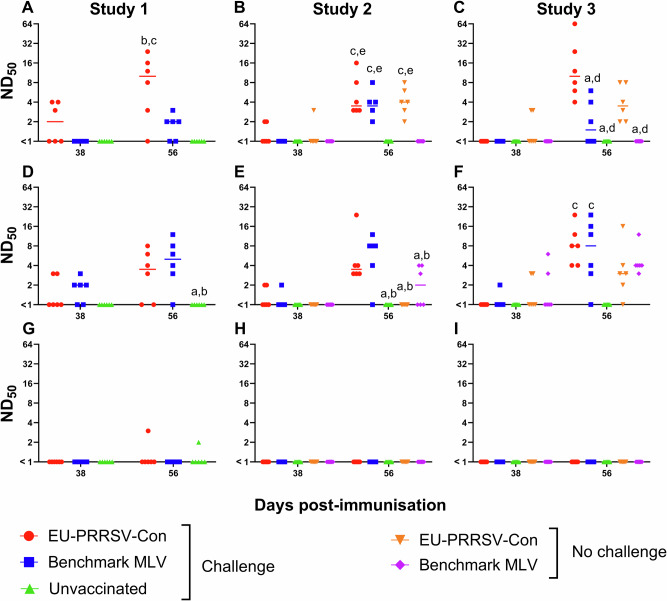
Assessment of PRRSV-neutralising antibody responses post-vaccination and -challenge. Serum samples from day 38 and 56 (3 and 21 DPC) were assessed for their ability to neutralise MARC-145 cell infection by vaccine viruses and BALC infection by challenge viruses. Neutralisation of EU-PRRSV-Con by study 1 (**A**), 2 (**B**) and 3 (**C**) sera; benchmark MLV by study 1 (**D**), 2 (**E**) and 3 (**F**) sera; 215-06 by study 1 sera (**G**); 21301-19 by study 2 sera (**H**); and SU1-Bel by study 3 sera (**I**) are shown with datapoints representing individual pig sera, and bars represent median values for each treatment group. Significances (*p* < 0.05) are described by letters where a: significant difference from EU-PRRSV-Con/challenged; b: significant difference from benchmark MLV/challenged; c: significant difference from unvaccinated/challenged; d: significant difference from EU-PRRSV-Con/no challenge; e: significant difference from benchmark MLV/no challenge.

PRRSV nAbs were evaluated in 6 paired serum samples/group collected on days 38 and 56 (3 and 21 DPC). Sera were assessed for their ability to neutralise MARC-145 cell infection by EU-PRRSV-Con and benchmark MLV viruses and BALC infection by the relevant challenge viruses, i.e. study 1 – 215-06, study 2 - 21301-19, and study 3 - SU1-Bel (Fig. 5). In study 1, at day 38, EU-PRRSV-Con nAbs were measurable in the serum of EU-PRRSV-Con immunised pigs (3/6) but not in sera from benchmark MLV vaccinated and unvaccinated pigs. At day 56, the EU-PRRSV-Con nAb titres were significantly higher in the EU-PRRSV-Con immunised group compared to both the benchmark MLV vaccinated and unvaccinated groups (*p* < 0.01) (Fig. 5A). In study 2, on day 38, EU-PRRSV-Con nAbs were only measurable in 2/6 of the serum of EU-PRRSV-Con immunised and in 1/6 of EU-PRRSV-Con immunised/challenged pigs and not in sera from the other groups (Fig. 5B). On day 56, EU-PRRSV-Con nAb titres were significantly greater in EU-PRRSV-Con groups and the benchmark MLV/challenged group compared to the unvaccinated/challenged and the benchmark MLV/unchallenged group (*p* < 0.05). In study 3, on day 38, EU-PRRSV-Con nAbs were only detected in the serum from 2/6 pigs in the EU-PRRSV-Con/unchallenged group. EU-PRRSV-Con nAb titres were significantly higher in the EU-PRRSV-Con/challenged and/unchallenged groups on day 56 compared to all other groups (*p* < 0.01) (Fig. 5C). In study 1, benchmark MLV nAbs were detected in 2/6 animals in the EU-PRRSV-Con and 4/6 of the benchmark MLV vaccinated on day 38. By day 56, benchmark MLV nAb titres were significantly higher in both vaccinated groups compared to the unvaccinated control group (*p* < 0.05) (Fig. 5D). For study 2, on day 38, benchmark MLV nAb titres were only detected in 2/6 and 1/6 pigs in the EU-PRRSV-Con and benchmark MLV immunised/challenged group, respectively. On day 56, nAb titres were significantly greater in both EU-PRRSV-Con and benchmark MLV vaccinated/challenged compared to the unvaccinated/challenged and the vaccinated/unchallenged groups (*p* < 0.05) (Fig. 5E). In study 3, benchmark MLV nAbs were only measured in the day 38 sera of 1/6 pigs in the benchmark MLV vaccinated/challenged group and 2/6 for both vaccinated/unchallenged groups. At day 56, both vaccinated/challenged groups had significantly higher benchmark MLV nAb titres compared to the unvaccinated/challenged group (*p* < 0.001) (Fig. 5F). Considering the capacity to neutralise the challenge strains, neutralisation of BALC infection by PRRSV-1 215-06 was only observed in sera from single animals in the EU-PRRSV-Con vaccinated and unvaccinated groups on day 56 (Fig. 5G). No sera neutralised PRRSV-1 21301-19 (Fig. 5H) nor SU1-Bel (Fig. 5I) infection of BALC.

### Immunisation with EU-PRRSV-Con elicits a robust T cell IFN-γ response

**Fig. 6:**
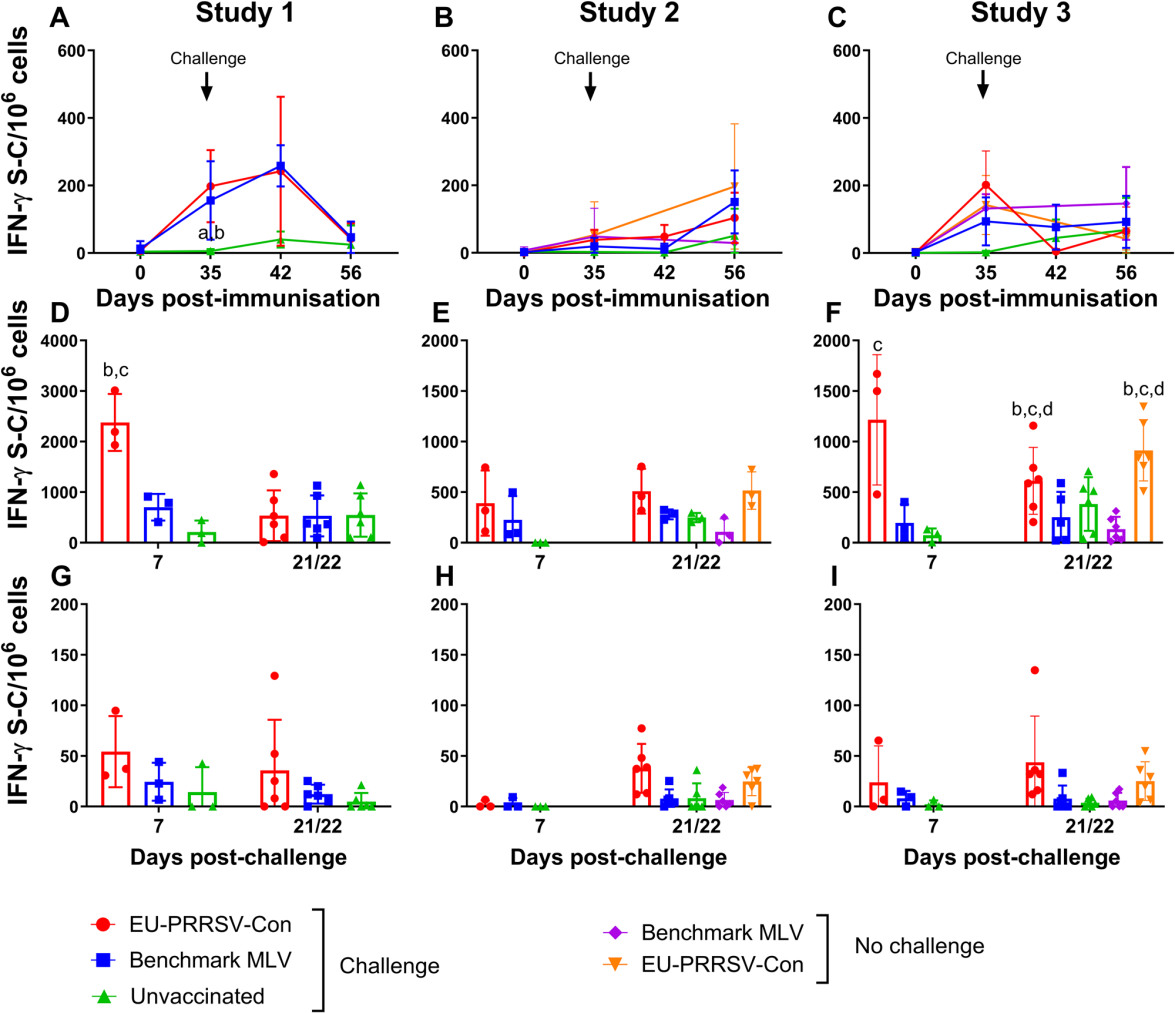
Assessment of PRRSV-specific IFN-γ responses post-vaccination and/or -challenge in PBMC, BALC and TBLN cells. Responses were assessed following PRRSV-1 stimulation of PBMC post-vaccination and/or -challenge in study 1 (**A**), 2 (**B**) and 3 (**C**), post-challenge (7 and 21/22 DPC) of BALC in study 1 (**D**), 2 (**E**) and 3 (**F**), and post-challenge (7 and 21/22 DPC) of TBLN cells in study 1 (**G**), 2 (**H**) and 3 (**I**). Datapoints represent mock-corrected data for individual pigs, line and bars represent the mean values for each treatment group, and error bars show the SD. Significances (*p* < 0.05) are described by letters where a: significant difference from EU-PRRSV-Con/challenged; b: significant difference from benchmark MLV/challenged; c: significant difference from unvaccinated/challenged; d: significant difference from EU-PRRSV-Con/no challenge; e: significant difference from benchmark MLV/no challenge.

IFN-γ ELISpot assays were performed on PRRSV-1 stimulated peripheral blood mononuclear cell (PBMC) samples isolated on day 0, 35, 42 and 56 across all three studies (Fig. 6). In study 1, PRRSV-1 specific IFN-γ responses were detected in both vaccinated groups at day 35 and 42, with responses in the EU-PRRSV-Con and benchmark MLV groups being significantly greater than the unvaccinated group (*p* < 0.01), however, responses did not differ significantly between groups by day 56 (Fig. 6A). In study 2 and 3, responses did not differ significantly between groups at any timepoint (Fig. 6B, C). Additionally, T cell responses were assessed by IFN-γ ELISpot assays with BALC isolated from pigs on day 42 and 55/56 (7 and 21/22 DPC) (Fig. 6D–F). In study 1 at 7 DPC, significantly higher IFN-γ responses were observed with BALC from EU-PRRSV-Con vaccinated/challenged pigs compared to both benchmark MLV vaccinated/challenged and unvaccinated/challenged pigs (*p* < 0.001) but groups did not differ significantly at 21/22 DPC (Fig. 6D). In study 2, responses were detected in BALC from vaccinated pigs at 7 DPC and all pigs at 21/22 DPC, but no significant differences were observed between groups, however there is a trend for higher responses in the EU-PRRSV-Con vaccinated animals (Fig. 6E). In study 3, at 7 DPC, BALC responses from the EU-PRRSV-Con vaccinated/challenged group were significantly greater than the unvaccinated/challenged group (*p* < 0.05). At 21/22 DPC, responses from both the EU-PRRSV-Con vaccinated/challenged and EU-PRRSV-Con vaccinated/unchallenged groups were significantly greater than all other groups (*p* < 0.05) (Fig. 6F). T cell responses were also assessed in TBLN cells isolated from the same time points described above (7 and 21/22 DPC) (Fig. 6G–I). Lower frequencies of PRRSV-specific IFN-γ secreting cells were detected in these samples compared to both PBMC and BALC, across all three studies. Although animals from EU-PRRSV-Con vaccinated/challenged demonstrated a trend for higher IFN-γ secreting cells across the studies at all time points evaluated, no significant differences were observed between groups at either time point in any of the studies.

## Discussion

The substantial antigenic diversity of circulating PRRSV strains poses a major challenge for control by vaccination. Current PRRS vaccines are failing to elicit sufficient levels of heterologous protection. Due to major advances in DNA synthesis and the subsequent manipulation of viral genomes, ‘reverse genetics’, numerous strategies have been developed in recent years to develop novel PRRSV vaccine candidates, including those based on consensus sequences^14,18,19,24^. This study applied the consensus sequence approach to ORFs 2–6 encoding the PRRSV-1 envelope proteins to develop a PRRSV-1 vaccine candidate, EU-PRRSV-Con. The ability of EU-PRRSV-Con to efficiently replicate in vitro in MARC-145 cells and porcine BALC cells was assessed, and the genetic stability of the virus was determined following sequential MARC-145 cell passage. The safety and immunogenicity of EU-PRRSV-Con and its ability to provide protection (reduction in clinical signs, lung pathology and viral loads) in pigs against heterologous challenge infections were assessed over three studies, benchmarking this against a widely used commercial PRRSV-1 MLV.

In the first study, pigs were challenged with the low virulent subtype 1 strain 215-06 and in the second study, with a higher virulence subtype 1 strain, 21301-19. As expected, no clinical signs were observed in any of the groups post-challenge and rectal temperatures remained stable, with no major differences between the groups, except for study 2, in which a short period of elevated rectal temperatures at 40- and 41-days post-immunisation was recorded in the benchmark MLV/challenged animals. This proved to be significantly higher than in the EU-PRRSV-Con challenged animals. However, as shown previously^25^, the virulent PRRSV-1 subtype 3 strain SU1-Bel, used in study 3, caused greater clinical signs and pyrexia compared to the PRRSV-1 subtype 1 strains. The clinical signs that were observed in study 3 were present in some of the benchmark MLV/challenged pigs, with no clinical signs observed in the EU-PRRSV-Con/challenged animals. Likewise, higher rectal temperatures were observed in all groups following SU1-Bel challenge than were recorded in the previous studies, again seeing higher temperatures in the benchmark MLV/challenged animals than the EU-PRRSV-Con/challenged group. In addition, infection of pigs with 21301-19 and SU1-Bel, respectively, induced greater gross lung pathology in the benchmark MLV/challenged animals than the EU-PRRSV-Con/challenged group. These results showed that whilst vaccination with EU-PRRSV-Con did not prevent challenge infection, it reduced the impact of clinical and pathological disease compared to those vaccinated with benchmark MLV. Furthermore, lower levels of viraemia and viral shedding (inferred by RT-qPCR) were detected in the EU-PRRSV-Con vaccinated group following challenge, in all studies. The results also demonstrated that the EU-PRRSV-Con groups had higher levels of viral RNA in serum and nasal swabs following immunisation compared to the benchmark MLV groups. These data were supported by the in vitro observations that EU-PRRSV-Con replicated efficiently in porcine BALC, as well as MARC-145 cells. In studies 1 and 2, it was found that significantly lower levels of virus were present in the BALF and lung tissue of the EU-PRRSV-Con/challenged animals compared to those that were unvaccinated, with there being significantly lower levels in the EU-PRRSV-Con group compared to the benchmark MLV group in study 1 and the complete absence of virus in the BALF from half of the EU-PRRSV-Con vaccinated animals. This further suggests that EU-PRRSV-Con is providing better protection than benchmark MLV against the subtype 1 PRRSV-1 strains. However, following challenge with the subtype 3 strain, significant differences were not detected between any of the vaccinated and unvaccinated groups. Of the PRRSV-1 strains that were used for the design of the EU-PRRSV-Con genome, the majority belong to Western European subtype 1 strains, with only one from subtype 2 and two from subtype 3 contributing to the ORF2–6 analysis, with an additional 15 subtype 2, 3 and 4 strains contributing to the ORF5 analysis. Therefore, as seen from the results of this study, the protective efficacy of EU-PRRSV-Con against subtype 3 strains and other divergent subtypes, such as those found in Eastern Europe, may be limited.

Previous studies have shown that nAbs play a significant role in providing immunity against PRRSV^26^. In a recently conducted study that developed a consensus sequence containing PRRSV-2 ORFs 2–6 based on 30 Chinese PRRSV strains, it was found that this consensus strain could induce broadly nAbs, whereas a conventional MLV strain induced only homologous antibodies^19^. This suggests that use of a consensus sequence strain, based on the PRRSV envelope glycoproteins, could be an effective strategy to induce broader nAbs that are protective against a diverse range of PRRSV strains. VNT assays were therefore performed on serum samples, assessing neutralisation of MARC-145 cell infectivity of the two vaccine strains and porcine macrophage infectivity of the respective challenge strain. Overall, immunisation with EU-PRRSV-Con resulted in enhanced nAb responses compared to benchmark MLV immunisation; with significantly greater neutralisation of EU-PRRSV-Con and comparable neutralisation of benchmark MLV, suggestive of a broader neutralisation. However, sera from both EU-PRRSV-Con and benchmark MLV immunised pigs were not able to neutralise challenge virus infection of macrophages. Whilst disappointing, this observation is frequently observed in studies of MLV induced antibody responses. Studies have shown that serum antibodies can more readily neutralise PRRSV infection of MARC-145 cells compared to infection of porcine macrophages^27–29^. Highlighting the need for a sensitive macrophage-based assay that can quantify the low titre neutralising antibodies elicited by PRRSV MLV and removing the need to adapt viruses to infection of MARC-145 cells.

The genetic stability of EU-PRRSV-Con was evaluated following sequential blind passaging (10 passages from P3) in MARC-145 cells. A limited number of SNPs were identified in the passage samples when compared to the original sequence. The identified SNPs predominantly affected non-structural proteins and would not affect the tropism of the virus or the antigenic properties of the envelope proteins. The lack of mutations found in the structural proteins is not unusual. A similar lack of SNPs in the structural proteins was found in an analysis of circulating PRRSV strains in Hong Kong^30^. The analysis of Hong Kong isolates also showed that most of the discovered SNPs had no effect on the protein sequence, which tallies with the data analysed here. An analysis of a PRRSV-1 inoculum revealed 16 major and 49 minor SNPs from the published sequence^31^. A high proportion of these changes were shown to be in nsp2 and within those, a majority of them were non-synonymous. This is comparable to the number of changes between the reference strain and in vitro samples seen in this study and their distribution across the genome. Whilst this degree of genetic stability in vitro is encouraging, studies are required to assess the potential for EU-PRRSV-Con to mutate to a virulent form following sequential passage in pigs (‘reversion to virulence’). Additional testing of the safety of EU-PRRSV-Con in pregnant sows and a more formal assessment of its shed and spread are warranted, especially given its high level of replication in immunised pigs.

In summary, the synthetic PRRSV-1 vaccine candidate, EU-PRRSV-Con, based on engineering consensus sequences encoding the envelope proteins (based on 67 PRRSV-1 full genome sequences mainly subtype 1, and an additional 15 ORF5 sequences from PRRSV-1 subtype 2, 3 and 4 strains) into the genome of an attenuated PRRSV-1 strain was successfully rescued, and evaluated both in vitro and in vivo. EU-PRRSV-Con was able to replicate high titres in cell culture in both MARC-145 cells and porcine macrophages. Growth curve analysis in MARC-145 cells showed efficient replication in line with that observed with the parental PRRSV-1 Olot/91 strain. Sequence analysis demonstrated that EU-PRRSV-Con was relatively stable after multiple passages in MARC-145 cells. EU-PRRSV-Con was found to be safe in pigs, with no adverse effects observed. EU-PRRSV-Con was immunogenic, inducing both T cell and nAb responses. Overall, EU-PRRSV-Con provided better levels of protection (reduced viral loads and lung lesions) than the licensed MLV, although the protective efficacy against the genetically divergent and pathogenic strain, SU1-Bel, was more limited. Collectively, these data support the further development of EU-PRRSV-Con as a vaccine that may improve the control of PRRSV-1.

## Methods

### Ethical approval

Animal studies were approved by the Animal and Plant Health Agency (APHA) and the Pirbright Institute Animal Welfare and Ethical Review Bodies and conducted under the Animals (Scientific Procedures) Act 1986 (Project Licence P6F09D691). Work with genetically modified PRRSV-1 was approved by the APHA Biological Safety Committee and the Pirbright Institute Biological Agents and Genetic Modification Safety Committee (Health and Safety Executive Notification Reference GM53).

### Cell culture

African green monkey kidney-derived MARC-145 cells were cultured and maintained in Dulbecco’s modified Eagle’s medium (DMEM) supplemented with 10% foetal bovine serum (FBS), 100 IU/mL penicillin, and 100 µg/mL streptomycin (all Thermo Fisher Scientific) (cDMEM). BHK-21 cells were cultured in Glasgow’s Minimum Essential Medium (GMEM BHK21) supplemented with 5% FBS, 5% Tryptose Phosphate Broth and 100 IU/mL penicillin, and 100 µg/mL streptomycin (all Thermo Fisher Scientific). Porcine BALC, PBMC and TBLN cells were cultured in RPMI-1640 medium supplemented with 10% FBS, 100 IU/mL penicillin, and 100 µg/mL streptomycin (all Thermo Fisher Scientific) (cRPMI). All cell lines were cultured at 37 °C with 5% CO_2_ in a humidified incubator.

### PRRSV-1 strains, propagation, and titration

PRRSV-1 strains used for immunisation were EU-PRRSV-Con (AC# PV146404) and Porcilis PRRS MLV (MSD Animal Health) (AC# MW674755, referred to here as ‘benchmark MLV’), whereas for challenge 215-06 (AC# OP047897^25^), 21301-19 (APHA, unpublished) and SU1-Bel AC# KP889243^25^) were used. PRRSV-1 Olot/91 (AC# KC862570^32^;) was used for the in vitro stimulation of PBMC and BALC. PRRSV-1 strains were propagated in MARC-145 cells (EU-PRRSV-Con, benchmark MLV, and Olot/91) or BALC (215-06, SU1-Bel, and 21301-19) by inoculating tissue culture flasks at a multiplicity of infection (MOI) of 0.01. The virus was harvested after culture at 37 °C for 3–4 days. To assess PRRSV infection, MARC-145 cells or BALC monolayers were subjected to immunoperoxidase staining and the median tissue culture infectious dose (TCID_50_) was calculated^33^.

### In silico design of EU-PRRSV-Con

Sequence data for ORFs 2–6 from PRRSV-1 strains were collated to a design consensus sequence. A selection of 67 genome sequences of PRRSV-1 strains was assembled, based on data published on GenBank® and data held at APHA (Supplementary Table 5). The majority of these strains belonged to subtype 1 (as previously defined^34^, but one subtype 2 and two subtype 3 strains were included. For ORF5 analyses, further sequences were added, to include seven from subtype 2, seven from subtype 3, and one from subtype 4 (Supplementary Table 6). From each full genome sequence, the specific ORF2, ORF2a, ORF3, ORF4, ORF5, ORF5a and ORF6 gene sequences were identified, and translated into the corresponding amino acid sequences. The resulting GP2, E, GP3, GP4, GP5, ORF5a protein, and M protein sequences were aligned using the Clustal W algorithm^35^. From these alignments, the consensus sequence was identified for each gene, based on the most prevalent amino acid at each position in the genes. The PRRSV-1 ESP-1991-Olot91 (Olot/91) strain (AC# KC862570)^36^ nucleotide sequence was used the backbone sequence for the EU-PRRSV-Con genome.

### Rescue of EU-PRRSV-Con

The process of rescuing EU-PRRSV-Con comprised four steps. Firstly, four overlapping DNA fragments covering the genome of EU-PRRSV-Con were synthesised by GeneArt Gene Synthesis Service (Thermo Fisher Scientific). The fragments were PCR amplified using Q5 high-fidelity Taq DNA polymerase (New England Biolabs) using specific primers (Table 2). PCR products were gel excised and purified using the GFX PCR DNA and Gel Band Purification Kit (Cytiva). Purified PCR products were transfected into BHK-21 strain 38 cells using TransIT-LT1 transfection reagent (Mirus Bio LLC). A clarified pool of culture supernatant and cell lysate from the transfected BHK-21 cell culture (Passage 0 - P0) was then passaged on MARC-145 cells. Once cytopathic effect (CPE) was observed, virus was titrated on MARC-145 cells as described previously.

**Table 2:** Primer sets to amplify DNA fragments spanning the EU-PRRSV-Con genome

Primer	Sequence 5′ to 3′
**Frag1 Forward**	GTTGACATTGATTATTGACTAGTT
**Frag1 Reverse**	GGGTCCTGAAAGCACCTTC
**Frag2 Forward**	TTCACACCATCTGCAGTTGG
**Frag 2 Reverse**	TTGCAGATATGGCCGGAGCT
**Frag 3 Forward**	CGTGTGCACTCTCACCGATG
**Frag 3 Reverse**	CAGCCAAGCATAATAACCCTCAAG
**Frag 4 Forward**	TGAGTTGTCTATGTCCATCCCGT
**Frag 4 Reverse**	AAGGCACAGTCGAGGCTGA

### Sequential passage and sequencing of EU-PRRSV-Con

Stability of EU-PRRSV-Con in cell culture was assessed by a serial passage of the virus on MARC-145 cells up to 10 passages. RNA was extracted from the tissue culture supernatants using the QIAamp Viral RNA mini kit (Qiagen) as per the manufacturer’s instructions. Passage 10 samples were prepared for sequencing on the MiSeq (Illumina) platform. The single-nucleotide polymorphism (SNP) analysis generated during the alignment was then used in Arraystar (Lasergene) for analysis of SNP position and function. MegAlign Pro (Lasergene) was used to align multiple samples and establish where SNPs were positioned. To be considered SNPs, the frequency of the change had to be above 25% in all aligned reads; for a major SNP, this was 50%, everything below 50% is considered a minor SNP. SNPs in non-coding regions were ignored.

### Vaccination and challenge studies

To evaluate the efficacy of EU-PRRSV-Con, three animal experiments, approved by The Pirbright Institute, Animal and Plant Health Agency and Ethics Committees, were conducted in accordance with the UK Animals (Scientific Procedures) Act 1986 (Project Licence P6F09D691). Studies were partially masked, i.e., blinded to the study/laboratory personnel responsible for recording or assessing clinical, pathological, virological, and immunological data. All studies were performed using PRRSV-naïve (antibody- and virus-free), large White-Landrace-Hampshire cross, female pigs, 6–8 weeks of age, sourced from a commercial, high health status farm, were randomly assigned to three treatment groups (*n* = 9). Within each treatment group, pigs were randomly allocated to be euthanized on day 49, 55, or 56 (*n* = 3 per timepoint). Group sizes were calculated based on PRRSV efficacy criteria (assessment of virus RNA levels) as described by ref. ^37^. Therefore, based on observed differences in Cq values between treated and untreated pigs, 6 pigs per group would allow the detection of difference between groups with 80% power and 95% confidence (power.t.test function in R (version 4.0.5)^38^). Animals were separated by pens with no direct contact between groups and were provided with water ad libitum and fed twice daily on a commercial diet ration.

As described in Table 1, on day 0, the first group was inoculated by intramuscular (i.m.) injection into the brachiocephalic muscle with 2 mL of the commercial PRRSV-1 MLV Porcilis PRRS® (MSD Animal Health), the second group with 1 mL phosphate buffered saline (PBS) containing 10^5^ TCID_50_ of PRRSV-1 EU-PRRSV-Con (P3) and the third group with 1 mL PBS. At 35 days post-immunisation, the three groups of pigs were challenged by intranasal inoculation with 2 mL (1 mL/nostril) 10^5^ TCID_50_ of PRRSV-1 subtype 1 strains 215-06 (study 1) and 21301-19 (study 2), and subtype 3 strain SU1-Bel (study 3) using a mucosal atomisation device (MAD 300, Wolfe Tory Medical, Inc, Salt Lake City, USA). Back titrations of both vaccine and challenge viruses were performed as described above to confirm doses administered. For studies 2 and 3, two additional groups (*n* = 6) were vaccinated but not challenged to assess vaccine virus in blood and tissues in the absence of challenge. Three pigs from challenged groups were euthanized on day 42 (7 DPC), and remaining pigs from all groups were euthanized days 55 and 56 (20 and 21 DPC, respectively). For euthanasia, animals were sedated by i.m. injection with a cocktail of Domitor (Medetomidine—1 mg/mL) and Zoletil (Tiletamine and Zolazepam—50 mg/mL) at a concentration of 0.5 mL/10 kg body weight, before an overdose of pentobarbital sodium anaesthetic (Pentoject—20 mL/animal) by intravenous injection in the marginal ear vein, followed by exsanguination, to enable post-mortem examination of lungs and collection of tissue samples.

### Clinical monitoring, pathological examination, and sample collection

Animals were clinically scored^37^, and rectal temperatures measured daily for 14 days after vaccination and challenge. Pigs were weighed weekly over the course of the study. Peripheral blood samples were collected into BD SST and heparin vacutainers (Thermo Fisher Scientific). For nasal swabs collections, cotton swabs (Scientific Laboratory Supplies, Nottingham, UK) were used and were placed into a specific media as described by De Brito et al.^37^. Following euthanasia, digital images of the dorsal and ventral aspects of the lungs were taken. The presence of gross lesions in each lung lobe was assessed by a veterinary pathologist (blinded as stated above) and scored semi-quantitatively to estimate the percentage of lung surface affected by pneumonia using a system adapted from Halbur et al.^39^. Two representative samples from each of the cranial, middle, and caudal lobes of the right lung (standardised location) were immersed in formalin 10% for histology and scored as described in ref. ^40^. Histopathological changes were assessed on lung tissue samples collected from pigs 7 DPC from the three studies. Lesions were scored blind using the “Morgan score”^41^, which evaluates the severity of necrosis of the bronchiolar epithelium, airway inflammation, perivascular/bronchiolar cuffing, alveolar exudates, and septal inflammation on H&E-stained sections. The “Iowa” scoring system^42^, which incorporates the presence of viral antigen, assessed by IHC, in the scoring, was also performed. Representative samples from the middle lobe of the right lung were frozen on dry ice for analysis by PCR. Bronchoalveolar lavage (BAL) was performed on the left lung using RPMI-1640 with 100 IU/mL penicillin, 100 µg/mL streptomycin, and 2% FBS (all reagents from Thermo Fisher Scientific) until 100 mL of BALF was obtained. TBLN were removed, and aliquots placed in Dulbecco’s PBS (DPBS) with 1% antibiotics and 2% FBS for cell isolation or snap frozen on dry ice for RT-qPCR analysis.

### Serum and cell isolation

Serum and PBMC were isolated as described by De Brito et al.^37^. BALF was obtained after centrifugation at 500 × *g* for 10 min, the supernatant was collected, and aliquots were frozen at −80 °C. The pellet was resuspended and washed twice in DPBS and filtered using a cell strainer (100 µm, Thermo Fisher Scientific) to obtain the BALC. TBLN were processed by chopping the tissue into fine pieces and dissociating cells by applying pressure of syringe barrel. Cells were filtrated as described for the BALC and erythrocytes lysed. PBMC, BALC and TBLN cells were resuspended in cRPMI or cryopreserved in 10% DMSO (Merck Life Science) in FBS.

### PRRSV-1 detection by quantitative RT-PCR

Lung and TBLN tissues were homogenised using M tubes and the gentleMACS™ Dissociator according to the manufacturer’s instructions (Miltenyi Biotec). Nasal swabs were vortexed in transport medium before removal and centrifugation of the swab fluid at 800 × *g* for 5 min at RT to remove any debris. RNA from serum, nasal swab supernatant, BALF, lung and TBLN homogenates was isolated using the MagMAX™-96 Viral RNA Isolation Kit and KingFisher™ Flex Purification System (both Thermo Fisher Scientific). The simple workflow was used for serum and nasal swab supernatants, whereas the complex workflow was used for BALF and tissue homogenates. The Exogenous Internal RNA Extraction Control was also included for all samples. RNA was eluted into a 96-well plate and RT-qPCR reactions were performed using the multiplex VetMAX™ PRRSV EU & NA 2.0 Kit (Thermo Fisher Scientific). Cq values were obtained in the 7500 Fast PCR system (Applied Biosystems™, Thermo Fisher Scientific) and Cq values below 40 were considered positive.

### IFN-γ ELISpot assay

PBMC, BALC or TBLN cells were plated at 2.5 × 10^5^ cells/well in 96-well PVDF membrane plates (Merck Life Science) coated with anti-porcine IFN-γ mAb (clone P2G10; BD Pharmingen). Cells were either left unstimulated (cRPMI) or stimulated with PRRSV-1 Olot/91 diluted to a multiplicity of infection of 0.1 in cRPMI, or with 5 µg/mL of concanavalin A as a positive control. All the conditions were performed in triplicate. Plates were incubated overnight at 37 °C and 5% CO_2_ and processed for IFN- γ staining as described by De Brito et al.^37^. Results were expressed as the number of IFN-γ-producing cells per 10^6^ cells minus the average number of IFN-γ-producing cells in mock-stimulated wells.

### PRRSV antibody ELISA

PRRSV specific antibodies were detected using a commercial diagnostic ELISA kit, PrioCHECK® PRRSV Ab porcine ELISA (Thermo Fisher Scientific). All the steps were described by the manufacturer and were followed accordingly^37^. After adding the stop solution, absorbance was measured at 450 nm and 620 nm using a spectrophotometer (GloMax^®^-Multi Detection System, Promega, Southampton, UK).

### PRRSV neutralisation assay

PRRSV neutralising titres were assessed by incubating serial 2-fold dilutions of heat-inactivated (56 °C for 30 min) sera with 400 TCID_50_ of PRRSV-1 for 1 h at 37 °C. Virus-serum mixtures were added to MARC-145 cells (1.5 × 10^4^ cells/well) or BALC (1.0 × 10^5^ cells/well) for vaccine (EU-PRRSV-Con or Porcilis) or challenge (215-06, 21301-19, or SU1-Bel) viruses, respectively. After incubated for 48 h at 37 °C with 5% CO_2_, immunoperoxidase staining using AEC substrate was conducted as described^37^. Neutralising antibody titres were calculated as the reciprocal serum dilution that fully neutralised viral infection in 50% of the wells (ND_50_).

### Statistical analysis

GraphPad Prism was used for graphical and statistical analysis of data sets. A two-way analysis of variance (ANOVA), followed by a Tukey’s multiple comparison post-hoc test, was employed. Differences were considered statistically significant when *p* < 0.05.

## Supplementary information

Supplementary Information

## Data Availability

The data that support the findings of this study are available from the corresponding author upon reasonable request. The EU-PRRSV-Con genomic sequence has been submitted to GenBank with ID: PV146404 and the sequence will be available upon publication. Genomic sequences for some of the strains used to design EU-PRRSV-Con have been submitted to GenBank: PRRSV-1 H2 strain ID: PV173709; PRRSV-1 87129-13 strain ID: PV173710; PRRSV-1 91110-14 ID: PV173711.

## References

[1] Lunney, J. K. et al. Porcine Reproductive and Respiratory Syndrome Virus (PRRSV): pathogenesis and interaction with the immune system. *Annu. Rev. Anim. Biosci.* **4**, 129–154 (2016). 26646630 10.1146/annurev-animal-022114-111025

[2] Wensvoort, G. et al. Mystery swine disease in the Netherlands: the isolation of Lelystad virus. *Vet. Q* **13**, 121–130 (1991). 1835211 10.1080/01652176.1991.9694296

[3] Mardassi, H., Wilson, L., Mounir, S. & Dea, S. Detection of porcine reproductive and respiratory syndrome virus and efficient differentiation between Canadian and European strains by reverse transcription and PCR amplification. *J. Clin. Microbiol.* **32**, 2197–2203 (1994). 7814546 10.1128/jcm.32.9.2197-2203.1994 PMC263966

[4] Vu, H. L. X., Pattnaik, A. K. & Osorio, F. A. Strategies to broaden the cross-protective efficacy of vaccines against porcine reproductive and respiratory syndrome virus. *Vet. Microbiol.* **206**, 29–34 (2017). 27692670 10.1016/j.vetmic.2016.09.014

[5] Kappes, M. A. & Faaberg, K. S. PRRSV structure, replication and recombination: Origin of phenotype and genotype diversity. *Virology* **479-480**, 475–486 (2015). 25759097 10.1016/j.virol.2015.02.012 PMC7111637

[6] Nan, Y. et al. Improved Vaccine against PRRSV: current Progress and Future Perspective. *Front. Microbiol.* **8**, 1635 (2017). 28894443 10.3389/fmicb.2017.01635 PMC5581347

[7] Montaner-Tarbes, S., Del Portillo, H. A., Montoya, M. & Fraile, L. Key Gaps in the Knowledge of the Porcine Respiratory Reproductive Syndrome Virus (PRRSV). *Front. Vet. Sci.* **6**, 38 (2019). 30842948 10.3389/fvets.2019.00038 PMC6391865

[8] Holtkamp, D. et al. Assessment of the economic impact of porcine reproductive 581 and respiratory syndrome virus on United States pork producers. *J. Swine Health Prod.* **21**, 72–84 (2013).

[9] Charerntantanakul, W. Porcine reproductive and respiratory syndrome virus vaccines: Immunogenicity, efficacy and safety aspects. *World J. Virol.* **1**, 23–30 (2012). 24175208 10.5501/wjv.v1.i1.23 PMC3782261

[10] Kim, H. et al. The assessment of efficacy of porcine reproductive respiratory syndrome virus inactivated vaccine based on the viral quantity and inactivation methods. *Virol. J.* **8**, 323 (2011). 21703032 10.1186/1743-422X-8-323 PMC3141684

[11] Bassaganya-Riera, J. et al. Impact of immunizations with porcine reproductive and respiratory syndrome virus on lymphoproliferative recall responses of CD8+ T cells. *Viral Immunol.* **17**, 25–37 (2004). 15018660 10.1089/088282404322875430

[12] Popescu, L. N., Trible, B. R., Chen, N. & Rowland, R. R. R. GP5 of porcine reproductive and respiratory syndrome virus (PRRSV) as a target for homologous and broadly neutralizing antibodies. *Vet. Microbiol* **209**, 90–96 (2017). 28528961 10.1016/j.vetmic.2017.04.016

[13] Stoian, A. M. M. & Rowland, R. R. R. Challenges for Porcine Reproductive and Respiratory Syndrome (PRRS) vaccine design: reviewing virus glycoprotein interactions with CD163 and targets of virus neutralization. *Vet Sci.* **6**, 10.3390/vetsci601000910 (2019). 10.3390/vetsci6010009 PMC6466263 30658381

[14] Vu, H. L. et al. A synthetic porcine reproductive and respiratory syndrome virus strain confers unprecedented levels of heterologous protection. *J. Virol.* **89**, 12070–12083 (2015). 26401031 10.1128/JVI.01657-15 PMC4645332

[15] Gaschen, B. et al. Diversity considerations in HIV-1 vaccine selection. *Science* **296**, 2354–2360 (2002). 12089434 10.1126/science.1070441

[16] Nickle, D. C. et al. Coping with viral diversity in HIV vaccine design. *PLoS Comput. Biol.* **3**, e75 (2007). 17465674 10.1371/journal.pcbi.0030075 PMC1857809

[17] Santra, S. et al. A centralized gene-based HIV-1 vaccine elicits broad cross-clade cellular immune responses in rhesus monkeys. *Proc. Natl. Acad. Sci. USA* **105**, 10489–10494 (2008). 18650391 10.1073/pnas.0803352105 PMC2483234

[18] Li, S. et al. A chimeric porcine reproductive and respiratory syndrome virus 1 strain containing synthetic ORF2-6 genes can trigger T follicular helper cell and heterologous neutralizing antibody responses and confer enhanced cross-protection. *Vet. Res* **55**, 28 (2024). 38449049 10.1186/s13567-024-01280-3 PMC10918997

[19] Chen, N. et al. Chimeric HP-PRRSV2 containing an ORF2-6 consensus sequence induces antibodies with broadly neutralizing activity and confers cross protection against virulent NADC30-like isolate. *Vet. Res.* **52**, 74 (2021). 34044890 10.1186/s13567-021-00944-8 PMC8161975

[20] Rahe, M. C. & Murtaugh, M. P. Mechanisms of Adaptive Immunity to Porcine Reproductive and Respiratory Syndrome Virus. *Viruses* **9**, 10.3390/v9060148 (2017). 10.3390/v9060148 PMC5490824 28608816

[21] Irwin, B., Heck, J. D. & Hatfield, G. W. Codon pair utilization biases influence translational elongation step times. *J. Biol. Chem.* **270**, 22801–22806 (1995). 7559409 10.1074/jbc.270.39.22801

[22] Xie, J. et al. A Triple Amino Acid Substitution at Position 88/94/95 in Glycoprotein GP2a of Type 1 Porcine Reproductive and Respiratory Syndrome Virus (PRRSV1) Is Responsible for Adaptation to MARC-145 Cells. *Viruses* **11**, 10.3390/v11010036 (2019). 10.3390/v11010036 PMC6356402 30626009

[23] Fang, Y. et al. Heterogeneity in Nsp2 of European-like porcine reproductive and respiratory syndrome viruses isolated in the United States. *Virus Res.* **100**, 229–235 (2004). 15019241 10.1016/j.virusres.2003.12.026

[24] Sun, H., Workman, A., Osorio, F. A., Steffen, D. & Vu, H. L. X. Development of a broadly protective modified-live virus vaccine candidate against porcine reproductive and respiratory syndrome virus. *Vaccine* **36**, 66–73 (2018). 29174314 10.1016/j.vaccine.2017.11.028

[25] Morgan, S. B. et al. Increased pathogenicity of European porcine reproductive and respiratory syndrome virus is associated with enhanced adaptive responses and viral clearance. *Vet. Microbiol.* **163**, 13–22 (2013). 23313323 10.1016/j.vetmic.2012.11.024

[26] Lopez, O. J. et al. Protection against porcine reproductive and respiratory syndrome virus (PRRSV) infection through passive transfer of PRRSV-neutralizing antibodies is dose dependent. *Clin. Vaccin. Immunol.* **14**, 269–275 (2007). 10.1128/CVI.00304-06 PMC1828847 17215336

[27] Robinson, S. R., Li, J., Nelson, E. A. & Murtaugh, M. P. Broadly neutralizing antibodies against the rapidly evolving porcine reproductive and respiratory syndrome virus. *Virus Res.* **203**, 56–65 (2015). 25845944 10.1016/j.virusres.2015.03.016

[28] Chen, R. et al. A porcine reproductive and respiratory syndrome virus (PRRSV)-specific IgM as a novel adjuvant for an inactivated PRRSV vaccine improves protection efficiency and enhances cell-mediated immunity against heterologous PRRSV challenge. *Vet. Res.* **53**, 65 (2022). 35986391 10.1186/s13567-022-01082-5 PMC9389807

[29] Liu, B. et al. Characterization of in vitro viral neutralization targets of highly pathogenic porcine reproductive and respiratory syndrome virus (HP-PRRSV) in alveolar macrophage and evaluation of protection potential against HP-PRRSV challenged based on combination of HP-PRRSV-structure proteins in vitro. *Vet. Microbiol.* **292**, 110035 (2024). 38484577 10.1016/j.vetmic.2024.110035

[30] Brar, M. S., Shi, M., Hui, R. K. & Leung, F. C. Genomic evolution of porcine reproductive and respiratory syndrome virus (PRRSV) isolates revealed by deep sequencing. *PLoS ONE* **9**, e88807 (2014). 24698958 10.1371/journal.pone.0088807 PMC3974674

[31] Lu, Z. H. et al. Quasispecies evolution of the prototypical genotype 1 porcine reproductive and respiratory syndrome virus early during in vivo infection is rapid and tissue specific. *Arch. Virol.* **162**, 2203–2210 (2017). 28361286 10.1007/s00705-017-3342-0 PMC5506507

[32] Mokhtar, H. et al. Proteome-wide screening of the European porcine reproductive and respiratory syndrome virus reveals a broad range of T cell antigen reactivity. *Vaccine* **32**, 6828–6837 (2014). 24844151 10.1016/j.vaccine.2014.04.054

[33] Hayhurst, E. et al. Evaluation of the delivery of a live attenuated porcine reproductive and respiratory syndrome virus as a unit solid dose injectable vaccine. *Vaccines* **10**, 1836 (2022). 36366345 10.3390/vaccines10111836 PMC9696641

[34] Stadejek, T. et al. Definition of subtypes in the European genotype of porcine reproductive and respiratory syndrome virus: nucleocapsid characteristics and geographical distribution in Europe. *Arch. Virol.* **153**, 1479–1488 (2008). 18592131 10.1007/s00705-008-0146-2

[35] Thompson, J. D., Higgins, D. G. & Gibson, T. J. CLUSTAL W: improving the sensitivity of progressive multiple sequence alignment through sequence weighting, position-specific gap penalties and weight matrix choice. *Nucleic Acids Res.* **22**, 4673–4680 (1994). 7984417 10.1093/nar/22.22.4673 PMC308517

[36] Kvisgaard, L. K. et al. A fast and robust method for full genome sequencing of Porcine Reproductive and Respiratory Syndrome Virus (PRRSV) Type 1 and Type 2. *J. Virol. Methods* **193**, 697–705 (2013). 23891870 10.1016/j.jviromet.2013.07.019

[37] de Brito, R. C. F. et al. An attenuated herpesvirus vectored vaccine candidate induces T-cell responses against highly conserved porcine reproductive and respiratory syndrome virus M and NSP5 proteins that are unable to control infection. *Front. Immunol.* **14**, 1201973 (2023). 37600784 10.3389/fimmu.2023.1201973 PMC10436000

[38] Team, R. C. R*:**A Language and Environment for Statistical Computing*, https://www.R-project.org (2021).

[39] Halbur, P. G., Paul, P. S., Vaughn, E. M. & Andrews, J. J. Experimental reproduction of pneumonia in gnotobiotic pigs with porcine respiratory coronavirus isolate AR310. *J. Vet. Diagn. Investig.* **5**, 184–188 (1993). 8389599 10.1177/104063879300500207

[40] Chrun, T. et al. Simultaneous infection with porcine reproductive and respiratory syndrome and influenza viruses abrogates clinical protection induced by live attenuated porcine reproductive and respiratory syndrome vaccination. *Front. Immunol.* **12**, 758368 (2021). 34858411 10.3389/fimmu.2021.758368 PMC8632230

[41] Morgan, S. B. et al. Pathology and virus distribution in the lung and lymphoid tissues of pigs experimentally inoculated with three distinct type 1 PRRS Virus Isolates of Varying Pathogenicity. *Transbound. Emerg. Dis.* **63**, 285–295 (2016). 25382098 10.1111/tbed.12272

[42] Gauger, P. C. et al. Kinetics of lung lesion development and pro-inflammatory cytokine response in pigs with vaccine-associated enhanced respiratory disease induced by challenge with pandemic (2009) A/H1N1 influenza virus. *Vet. Pathol.* **49**, 900–912 (2012). 22461226 10.1177/0300985812439724

