# Supplementary material for: A centralised immunogen approach to develop a more broadly protective modified live porcine reproductive and respiratory syndrome virus 1 vaccine candidate
Source: NPJ Vaccines. 2025 Jun 21;10:129. doi: 10.1038/s41541-025-01192-z (PMC12182563; doi:10.1038/s41541-025-01192-z)
Supplement: Supplementary file 1 — Supplementary Information [file 41541_2025_1192_MOESM1_ESM.pdf]

A. PRRSV-1 GP2 alignment showing consensus strength at each position:

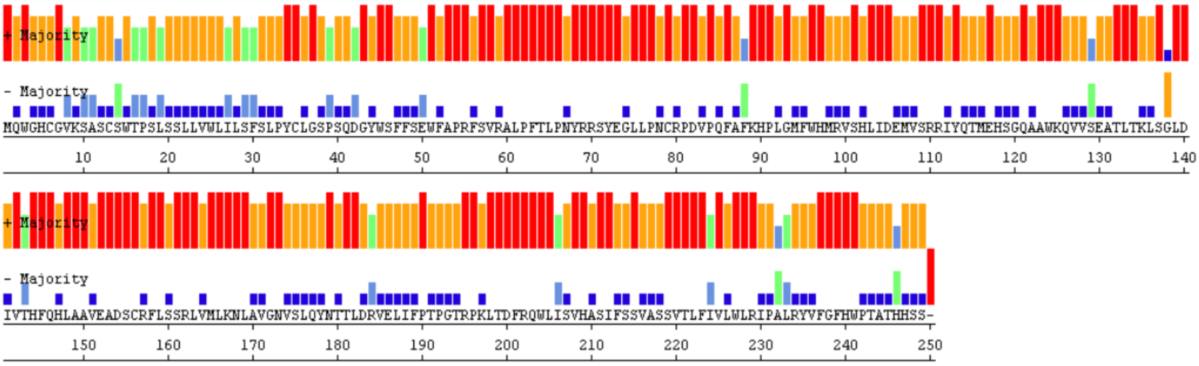

B. PRRSV-1 E alignment showing consensus strength at each position:

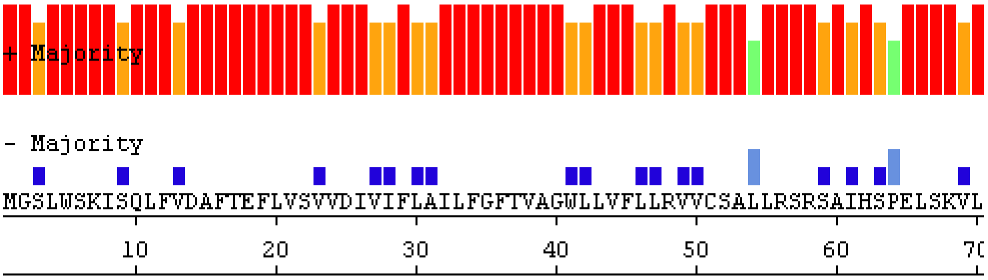

C. PRRSV-1 GP3 alignment showing consensus strength at each position:

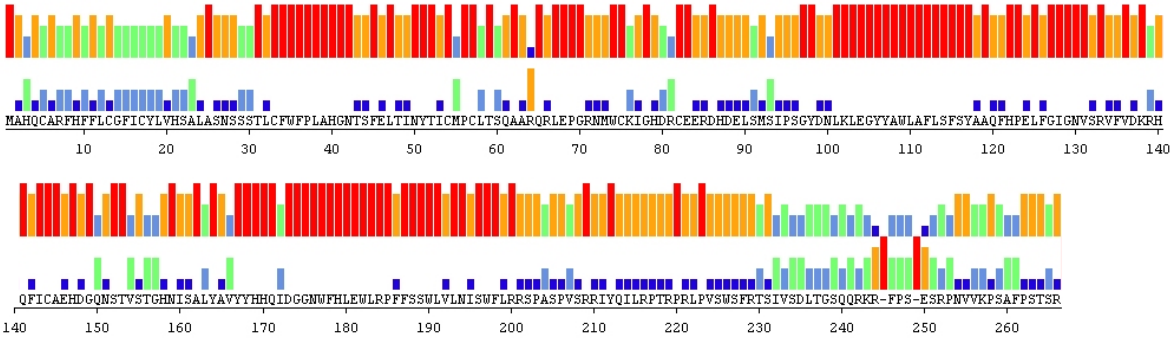

D. PRRSV-1 GP4 alignment showing consensus strength at each position:

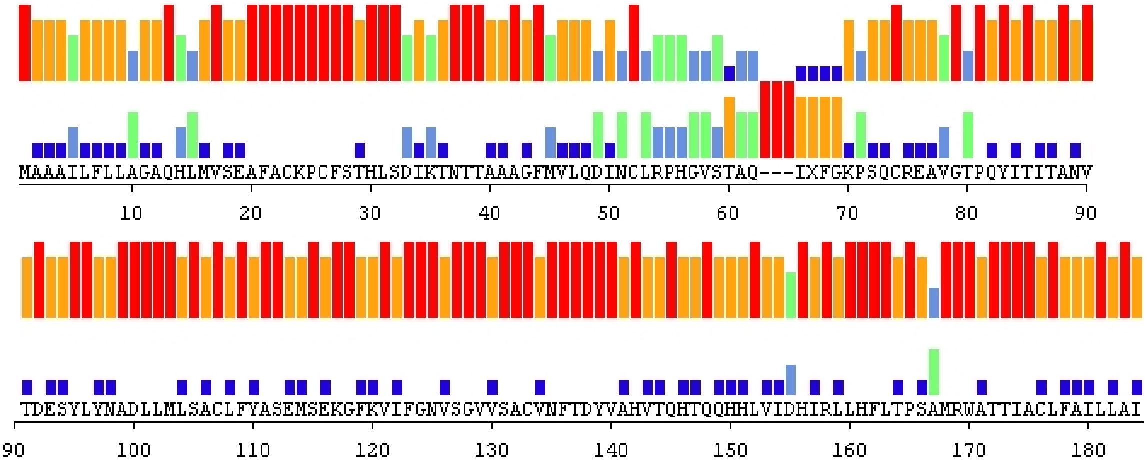

E. PRRSV-1 GP5 alignment showing consensus strength at each position:

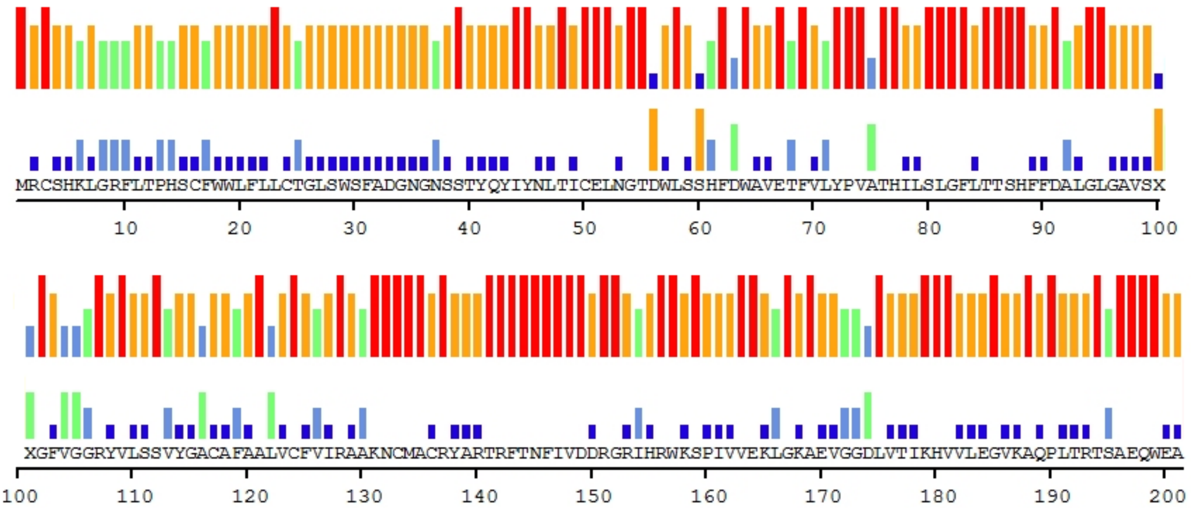

F. PRRSV-1 GP5a alignment showing consensus strength at each position:

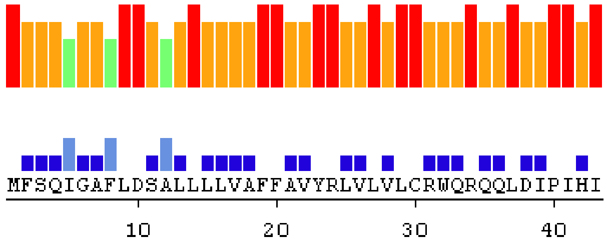

G. PRRSV-1 M alignment showing consensus strength at each position:

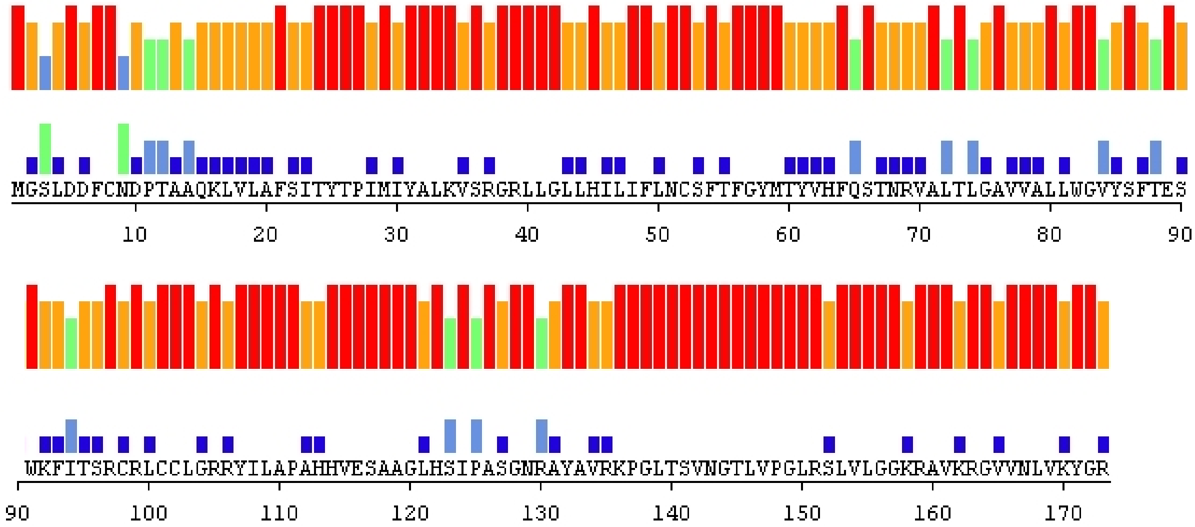

Supplementary Figure 1. Consensus sequences for EU-PRRSV-Con structural proteins, showing consensus strength at each position. Analysis conducted using MegAlign v.13 (DNASTAR©). **A-G** represents GP2, E, GP3, GP4, GP5, GP5a, and M protein, respectively.

Supplementary Figure 2. Phylogenetic relationship of EU-PRRSV-Con and other PRRSV-1 strains._Evolutionary history was inferred using the Neighbour-Joining method (Saitou and Nei, 1987). The evolutionary distances were computed using the Kimura 2-parameter method (Kimura, 1980) and are in the units of the number of base substitutions per site. The rate variation among sites was modelled with a gamma distribution (shape parameter = 0.66). The analysis involved 73 nucleotide sequences, including one reference PRRSV-2 strain. These analyses were conducted in MEGA7. The EU-PRRSV-Con sequence is shown by a red marker, challenge strains by blue markers and current commercial MLV vaccine strains by green markers.

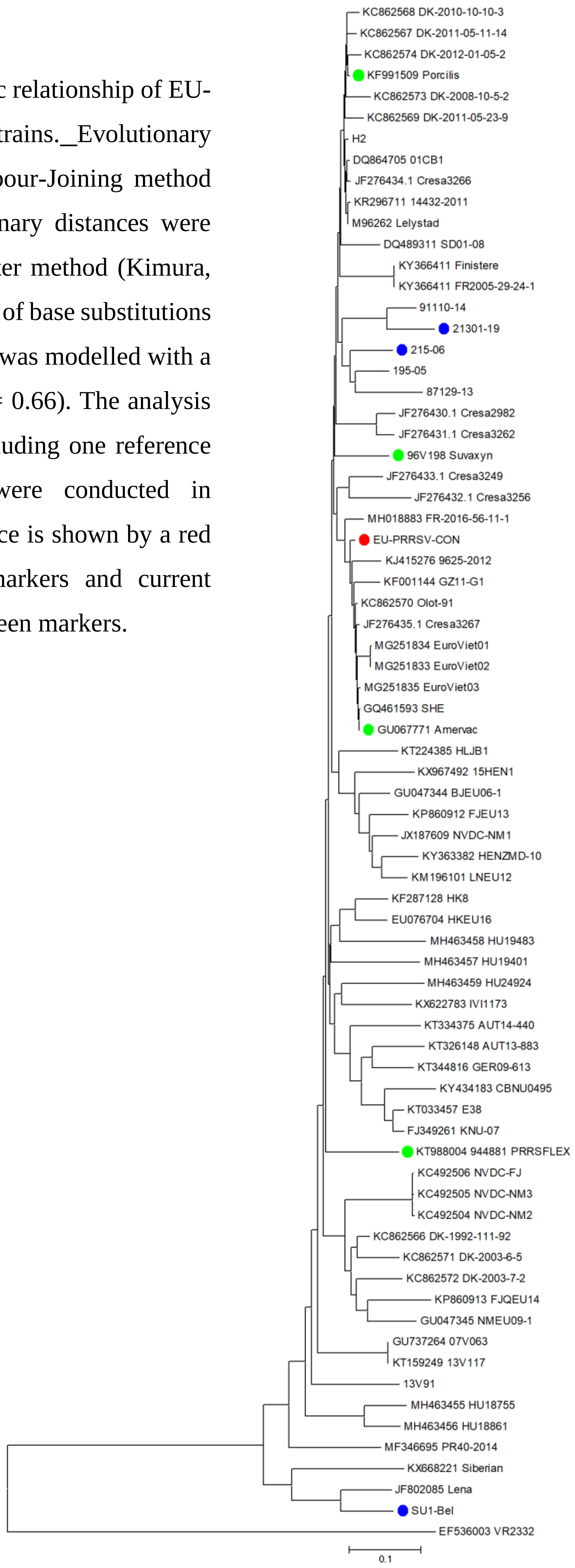

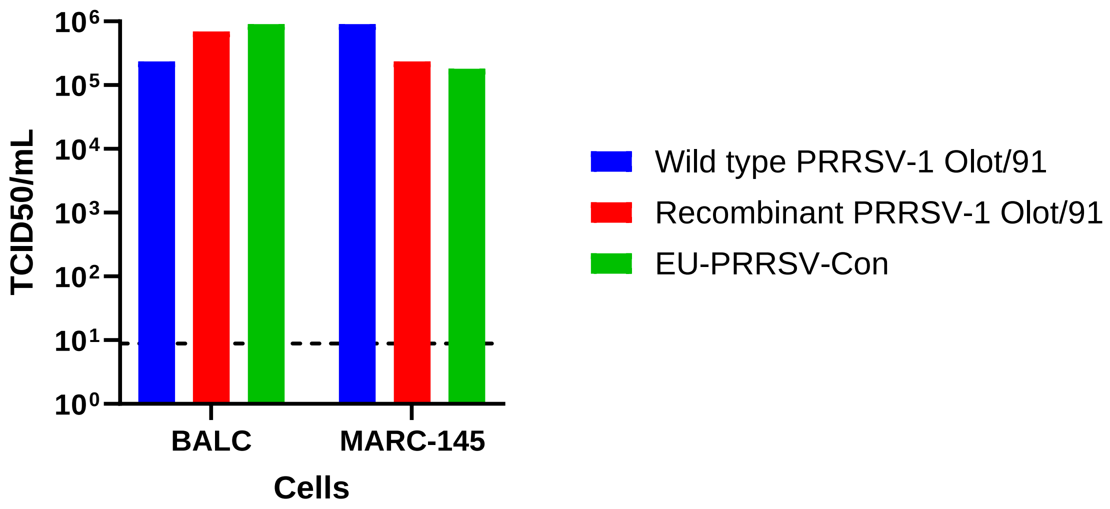

Supplementary Figure 3. Assessment of EU-PRRSV-Con replication in MARC-145 cells and porcine BALC. MARC-145 propagated stocks of EU-PRRSV-Con, wild type and recombinant PRRSV-1 Olot/91 were titrated in MARC-145 cells and BALC. The dashed horizontal line indicates the assay limit of detection.

Supplementary Table 1. Single-nucleotide polymorphisms in in vitro passaged samples of EU-PRRSV-Con (R1, R2 and R3). SNP defined by frequency of the change in >25% of aligned reads. Major SNP defined as occurring in >50% of aligned reads and their number shown in parentheses.

	**R1**			**R2**			**R3**		
Total SNPs	23			30			18		
Synonymous	12			21			12		
Non-synonymous	2			9			6		
	Total	Synonymous	Non-synonymous	Total	Synonymous	Non-synonymous	Total	Synonymous	Non-synonymous
pp1a	4 (2)	2 (2)	2 (0)	8 (1)	3 (1)	5 (0)	4 (3)	1 (1)	3 (2)
pp1b	14 (13)	11 (10)	3 (3)	15 (10)	15 (10)	0 (0)	10 (10)	9 (9)	1 (1)
GP2	1 (1)	1 (1)	0 (0)	2 (1)	1 (1)	1 (0)	1 (1)	1 (1)	0 (0)
Envelope	0	0	0	0	0	0	0	0	0
GP3	1 (0)	0 (0)	1 (0)	2 (0)	1 (0)	1 (0)	1 (0)	0 (0)	1 (0)
GP4	1 (0)	0 (0)	1 (0)	1 (0)	0 (0)	1 (0)	1 (0)	0 (0)	1 (0)
GP5	1 (0)	1 (0)	0 (0)	1 (0)	1 (0)	0 (0)	1 (1)	1 (1)	0 (0)
ORF5a	0	0	0	1 (0)	0 (0)	1 (0)	0	0	0
Membrane	1 (1)	1(1)	0 (0)	0	0	0	0	0	0
Nucleocapsid	0	0	0	0	0	0	0	0	0

Supplementary Table 2. Percentage identity between the EU-PRRSV-Con and Benchmark MLV compared to the three PRRSV-1 challenge viruses for both the complete genome (nucleotide only) and ORFs 2-6 (nucleotide and amino acid).

	**Vaccine**					
	**EU-PRRSV-Con**			**Benchmark MLV**		
	**Challenge strains**					
	**215-06**	**21301-19**	**SU1-Bel**	**215-06**	**21301-19**	**SU1-Bel**
**Nucleotide % identity**						
**Complete genome**	91.2	87.3	83.0	92.8	88.5	82.8
**ORF2a**	92.1	91.0	85.8	93.2	92.0	84.8
**ORF3**	90.7	89.4	85.2	90.6	89.3	82.2
**ORF4**	88.7	87.0	82.7	88.0	87.2	81.7
**ORF5**	94.2	90.1	83.0	93.1	89.1	83.4
**ORF6**	93.5	93.2	86.0	94.4	94.3	88.5
**Amino acid % identity**						
**GP2**	92.8	93.6	87.6	90.8	92.0	85.5
**GP3**	88.6	88.5	79.7	87.2	87.0	77.8
**GP4**	90.1	90.7	83.2	87.4	89.1	82.1
**GP5**	94.5	91.0	85.1	92.0	89.6	84.6
**M**	96.0	96.5	94.2	96.5	96.0	93.6

Supplementary Table 3. Back-titration of inoculum to confirm of vaccine and challenge virus doses.

**Virus**	**Titre ($TCID_{50}$)/dose**		
	**Study 1**	**Study 2**	**Study 3**
Porcilis PRRS (MSD Animal Health)	1.72×10^5	5.44×10^5	8.00×10^5
EU-PRRSV-Con	1.35×10^5	6.32×10^4	6.32×10^4
PRRSV-1 215-06	1.33×10^5	n/a	n/a
PRRSV-1 21301-19	n/a	5.91×10^5	n/a
PRRSV-1 SU1-Bel	n/a	n/a	9.66×10^4

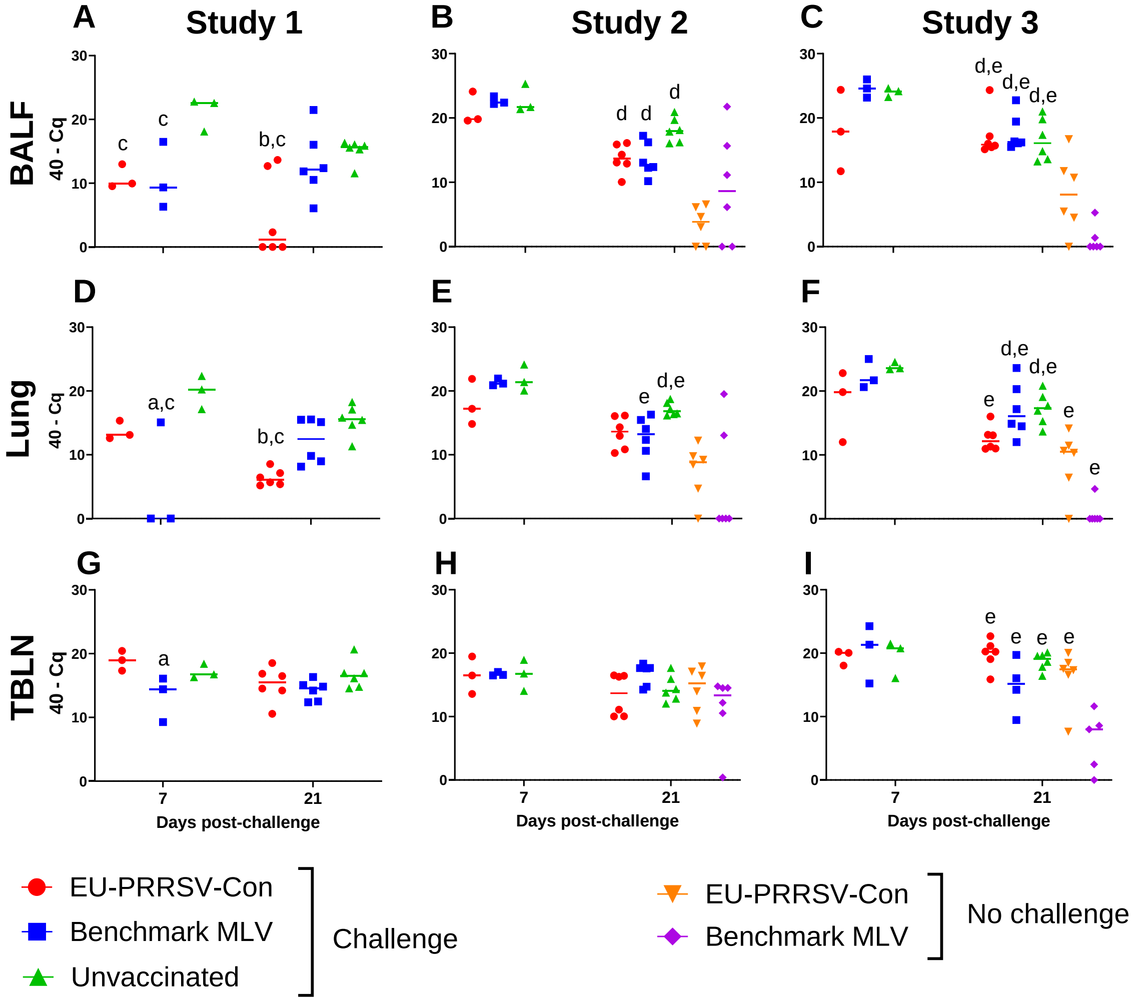

Supplementary Figure 6. Viral loads present in tissues of each group post-challenge. On 7 days post-challenge (day 42), 3 pigs from each of the challenge groups were euthanised and BALF (A), lung (B) and TBLN (C) tissues collected and assessed for viral loads using RT-qPCR. On the termination of the studies 21- and 22-days post-challenge (day 56 and 57), the remaining pigs were euthanised and tissues analysed. Results are expressed as 40-Cq value for each pig at 7- and 21/22-days post-challenge and bars represent median values for each treatment group.

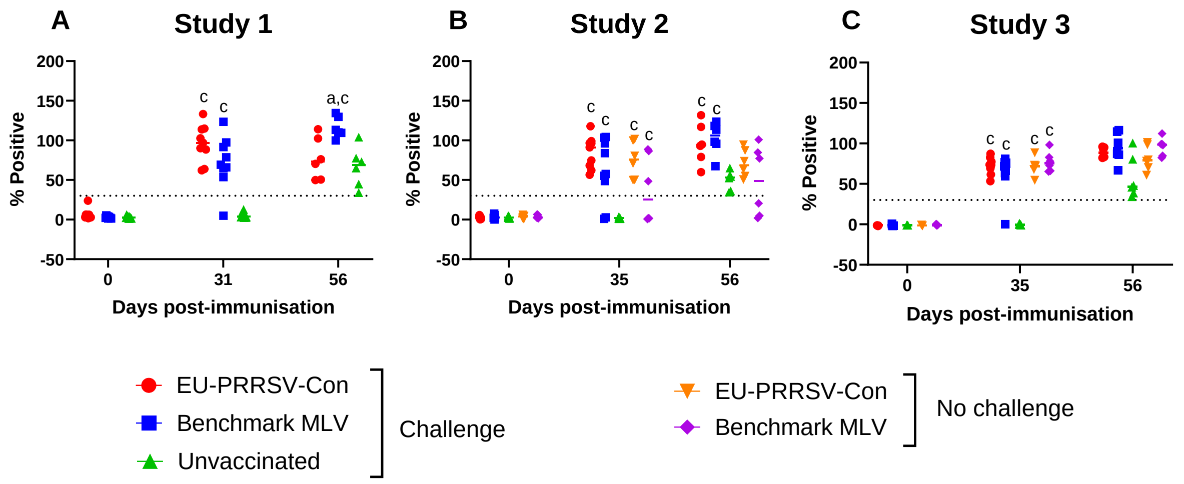

Supplementary Figure 7. Assessment of PRRSV-specific antibody responses post-vaccination and -challenge. Serum samples were assessed for PRRSV-specific antibodies by ELISA and data presented as percent positive relative to the corrected OD 450 nm max. Results obtained above 30% positive (dashed horizontal line) are considered positive. Data for each pig sera are shown, and bars represent median values for each treatment group.

Supplementary Table 4. Comparative evaluation of the area under the curve (AUC) for PRRSV RNA levels in serum and nasal swabs during pre-challenge and post-challenge periods.

Parameter	Period	Study	Area under the curve (95% Confidence Interval)		
			EU-PRRSV-Con/challenged	Benchmark MLV/challenged	Unvaccinated/challenged
Viraemia (RT-qPCR)	Pre-challenge	1	390.8 (346.8 to 434.8)	203.7 (140.2 to 267.1)	0 (0 to 0)
		2	450.9 (400.3 to 501.5)	173 (64.34 to 281.6)	0 (0 to 0)
		3	451.8 (401.5 to 502.2)	289.9 (198.8 to 381.0)	0 (0 to 0)
	Post-challenge	1	66.1 (53.23 to 158.5)	105.9 (27.02 to 105.2)	220.4 (188.8 to 252.1)
		2	129.4 (83.70 to 175.0)	224.7 (165.3 to 284.0)	258 (234.5 to 281.5)
		3	209.5 (167.9 to 251.1)	268.7 (227.1 to 310.3)	320.8 (299.5 to 342.2)
Nasal shedding (RT-qPCR)	Pre-challenge	1	40.77 (11.03 to 70.51)	20.02 (0.000 to 48.77)	0 (0 to 0)
		2	33.16 (0.000 to 80.76)	5.374 (0.000 to 26.57)	0 (0 to 0)
		3	30.1 (0.000 to 71.14)	23.95 (0.000 to 59.71)	0 (0 to 0)
	Post-challenge	1	9.73 (0.000 to 28.11)	13.81 (0.000 to 32.82)	34.1 (6.280 to 61.91)
		2	31.4 (0.000 to 68.45)	64.49 (25.70 to 103.3)	51.2 (22.73 to 79.66)
		3	55.08 (14.50 to 95.66)	98.98 (52.72 to 145.2)	130.6 (103.5 to 157.7)

Supplementary Table 5. PRRSV-1 genome sequences used to design the consensus sequence.

#	Strain	GenBank® AC#	Subtype	Origin
1	01CB1	DQ864705	1	Thailand
2	07V063	GU737264	1	Belgium
3	13V117	KT159249	1	Belgium
4	13V91	KT159248	1	Belgium
5	14432-11	KR296711	1	Hungary
6	15HEN1	KX967492	1	China
7	195-05	KU560579	1	UK
8	215-06	OP047897	1	UK
9	87129-13	PV173710	1	UK
10	91110-14	PV173711	1	UK
11	94881	KT988004	1	USA
12	9625/2012	KJ415276	1	Hungary
13	AUT13-883	KT326148	1	Austria
14	AUT14-440	KT334375	1	Austria
15	BJEU06-1	GU047344	1	China
16	CBNU0495	KY434183	1	Korea
17	Cresa2982	JF276430	1	Spain
18	Cresa3249	JF276433	1	Spain
19	Cresa3256	JF276432	1	Spain
20	Cresa3262	JF276431	1	Spain
21	Cresa3266	JF276434	1	Germany
22	Cresa3267	JF276435	1	Portugal
23	DK-1992-111-92	KC862566	1	Denmark
24	DK-2003-6-5	KC862571	1	Denmark
25	DK-2003-7-2	KC862572	1	Denmark
26	DK-2008-10-5-2	KC862573	1	Denmark
27	DK-2010-10-10-3	KC862568	1	Denmark
28	DK-2011-05-11-14	KC862567	1	Denmark
29	DK-2011-05-23-9	KC862569	1	Denmark
30	DK-2012-01-05-2	KC862574	1	Denmark
31	E38	KT033457	1	Korea
32	EuroViet-01	MG251834	1	Vietnam
33	EuroViet-02	MG251833	1	Vietnam
34	EuroViet-03	MG251835	1	Vietnam
35	Finistère	KY366411	1	France

36	FJEU13	KP860912	1	China
37	FJQUEU14	KP860913	1	China
38	FR-2005-29-24-1	KY366411	1	France
39	FR-2016-56-11-1	KY767026	1	France
40	GER09-613	KT344816	1	Germany
41	GZ11-G1	KF001144	1	China
42	H2	PV173709	1	UK
43	HENZMD-10	KY363382	1	China
44	HK8	KF287128	1	Hong Kong
45	HKEU16	EU076704	1	Hong Kong
46	HLJB1	KT224385	1	China
47	HU18755/2016	MH463455	1	Hungary
48	HU18861/2016	MH463456	1	Hungary
49	HU19401/2016	MH463457	1	Hungary
50	HU19483/2016	MH463458	1	Hungary
51	HU24924/2016	MH463459	1	Hungary
52	IVI-1173	KX622783	1	Switzerland
53	KNU-07	FJ349261	1	Korea
54	Lelystad	M96262	1	Netherlands
55	Lena	EU909693	3	Belarus
56	LNEU12	KM196101	1	China
57	NMEU09-1	GU047345	1	China
58	NVDC-FJ	KC492506	1	China
59	NVDC-NM1	JX187609	1	China
60	NVDC-NM2	KC492504	1	China
61	NVDC-NM3	KC492505	1	China
62	Olot91	KC862570	1	Spain
63	PR40/2014	MF346695	1	Italy
64	SD01-08	DQ489311	1	USA
65	SHE	GQ461593	1	China
66	Siberian	KX668221	2	Russia
67	SU1-Bel	KP889243	3	Belarus

Supplementary Table 6. Additional PRRSV-1 ORF5 sequences used to design the consensus sequence.

#	**Strain**	**GenBank® AC#**	**Subtype**	**Origin**
1	Bel-42	DQ324669	3	Belarus
2	BK	EU071231	2	Russia
3	BLG	EU071232	2	Russia
4	Bor	JN651734	2	Belarus
5	BT-2	EU071247	2	Russia
6	ND-3	EU071249	2	Russia
7	Obu	DQ324676	3	Belarus
8	OKT	JN651736	4	Belarus
9	Soz-6	DQ324686	3	Belarus
10	Soz-f2	EU071227	3	Belarus
11	Soz-f3	EU071228	3	Belarus
12	VL-3	EU071246	2	Russia
13	Vos	DQ324690	3	Belarus
14	Zad-1	DQ324694	3	Belarus
15	ZV	EU071245	2	Russia
